# Facilitating HIV/AIDS and HIV testing literacy for emergency department patients: a randomized, controlled, trial

**DOI:** 10.1186/s12873-018-0172-7

**Published:** 2018-07-09

**Authors:** Roland C. Merchant, Tao Liu, Melissa A. Clark, Michael P. Carey

**Affiliations:** 1 Department of Emergency Medicine, Brigham and Women’s Hospital, Harvard Medical School, 75 Francis Street, Boston, MA 02115 USA; 2 Department of Biostatistics, Center for Statistical Sciences, School of Public Health, Brown University, Providence, RI 02906 USA; 3 Department of Quantitative Health Sciences, University of Massachusetts Medical School, 368 Plantation Street, Worcester, MA 01605 USA; 4 Centers for Behavioral and Preventive Medicine, The Miriam Hospital, Coro West, Suite 309, 164 Summit Ave, Providence, RI 02906 USA; 5 Department of Psychiatry and Human Behavior, Alpert Medical School, Brown University, Providence, RI 02906 USA

**Keywords:** HIV, Emergency medicine, Emergency department, Health literacy, Randomized controlled trial

## Abstract

**Background:**

Although this has not been fully studied, videos and pictorial brochures might be equivalent methods of delivering HIV/AIDS and HIV testing information to emergency departments (ED) patients. It also is not known how well or for how long such knowledge is retained, if this information should be tailored according to patient health literacy, and if retention of this knowledge impacts future HIV testing behavior.

**Methods:**

We will conduct a multi-site, randomized, controlled, longitudinal trial among 600 English- and 600 Spanish-speaking 18–64-year-old ED patients to investigate these questions. We will stratify our sample within language (English vs. Spanish) by health literacy level (lower vs. higher) and randomly assign patients to receive HIV/AIDS and HIV testing information by video or pictorial brochure. All patients will be tested for HIV in the ED. At 12-months post-enrollment, we will invite participants to be tested again for HIV. As primary aims, we will compare the efficacy of pictorial brochures and videos in improving short-term (in ED) HIV/AIDS and HIV testing knowledge and retaining this knowledge over 12 months. We will determine if and how short-term improvement and longer-term retention of knowledge interacts with information delivery mode (pictorial brochure or video), patient health literacy level (lower or higher), and language (English or Spanish). As secondary aims, using the Information-Motivation-Behavioral Skills (IMB) model as a heuristic framework, we will measure constructs from the IMB model relevant to our study, and assess their impact on HIV re-testing behavior; we will also examine the moderating influences of information delivery mode, language, and health literacy level. In addition, we will explore simplified screening strategies to identify ED patients with lower health literacy as ways to implement a tailored approach to HIV/AIDS and HIV testing information delivery in EDs.

**Discussion:**

Study findings will guide ED-based delivery of HIV/AIDS and HIV testing information; that is, whether delivery modes (video or pictorial brochure) should be selected for patients by language and/or health literacy level. The results also will inform EDs when, how, and for whom information needs to be provided for those undergoing testing again for HIV within a one-year period.

**Trial registration:**

ClinicalTrials.gov Identifier: NCT02284451. Posted November 6, 2014.

**Electronic supplementary material:**

The online version of this article (10.1186/s12873-018-0172-7) contains supplementary material, which is available to authorized users.

## Background

The United States (US) Centers for Disease Control and Prevention (CDC) recommends that all patients at every HIV testing encounter receive information orally or in writing about HIV/AIDS and HIV testing [1]. CDC recommends that this information includes the benefits and consequences of HIV testing, HIV transmission and prevention, testing methods/procedures, the meaning of possible test results, and how to find additional information for counseling and other services [2]. The primary intent of this information is to improve HIV/AIDS and HIV testing knowledge, although it likely also affects motivation and behavioral skills around HIV testing, HIV risk-taking behaviors, and future HIV testing utilization. However, no published research exists that indicates how, in what form, and how often this information should be delivered to improve and retain HIV/AIDS and HIV testing knowledge; if it is best delivered by the two CDC-recommended delivery modes (orally or in writing); if this information must be provided to all patients at every testing encounter; if and how it could be delivered according to patient needs and abilities (e.g., health literacy skills); or how this information and its delivery mode affects patients HIV testing motivation and skills, HIV risk-taking, and future HIV re-testing*.*

Oral information delivery is limited by variations in staff abilities, access to translators, and scarce resources. Evidence supporting the efficacy of written informational brochures in improving HIV/AIDS and HIV testing knowledge is scant*.* A 1986 study reported that only 40% of Swiss citizens receiving an informational brochure about HIV/AIDS read it entirely and 11% refused to read it, yet those who read it had greater HIV/AIDS knowledge 2 weeks later [3]. A United Kingdom sexually transmitted disease (STD) clinic study found that an informational pamphlet increased HIV testing knowledge similar to an in-person discussion for lower HIV risk clients [4]. However, written brochures might not be useful for those with lower health or general literacy, especially when in a text-only form [5–8]. A study of 136 HIV/AIDS written materials found that more than half were at a 10th–12th-grade reading comprehension level and 13% were at a university level [5]. As such, these materials are not useful for lower literacy patients. Of greater concern, a 1997 review of 72 state, federal, or privately-produced HIV/AIDS brochures concluded that approximately 95% contained inaccuracies so severe that their use was not recommended [9].

Having efficacious yet efficient modes of delivering HIV/AIDS and HIV testing information can remove a barrier to emergency department (ED) HIV testing. For EDs, videos might be a better alternative to oral and written HIV/AIDS and HIV testing information*.* They can be delivered at any time or location with fewer staffing requirements; can be provided to those awaiting services, making the testing encounter more efficient; and can be adapted for multiple languages, which helps reduce the need for in-person or telephonic translation services. Videos might be better than written brochures because they combine audio and visual imagery that can work together to enhance the effectiveness of delivering information [10, 11], especially for people with lower general literacy and lower health literacy [12, 13]. ED-based, video-delivered HIV/AIDS and HIV testing information has been promising. Calderon, et al. observed that ED patients watching a “pre-test” HIV informational video scored better on a 10-item knowledge questionnaire than those receiving information orally (∆ 5.6%; 90% CI 2.6–8.7%) [14]. In addition, adult ED patients watching a “post-test” video scored better than those in an oral information group (∆ 6.9%; 90% CI 2.8–11.2%) [15], as did pediatric/young adult ED patients (∆ 12.2%; 95% CI 3.2–16.5%) [16].

Our research group has conducted several studies examining delivery of HIV/AIDS and HIV testing information in EDs. In a 2005 pilot study, English-speaking, 18–55-year-old Rhode Island Hospital ED patients were randomly assigned to receive either no information or information delivered orally by an HIV test counselor (a research assistant [RA]) [17]. The topics for the orally-delivered information reflected CDC recommendations on HIV/AIDS and HIV testing information that HIV test recipients should receive when being tested for HIV [2, 18]. All study participants completed a 26-item questionnaire to measure their HIV/AIDS and HIV testing knowledge applicable to these CDC recommendations. Participants randomly assigned to the no information arm of the pilot study answered an average of 50% of the questions correctly, compared to an average of 77% who received information orally (*p* < 0.001). These results indicated a need for HIV/AIDS and HIV testing information for ED patients.

During 2004–2005, we produced a professional quality, animated and live-action, 9.5 min, English-language HIV/AIDS and HIV testing informational video, “Do you know about rapid HIV testing?” [17] During 2005–2006, we conducted a randomized, controlled, non-inferiority trial (K23AI060363) to determine if the video was as efficacious in improving HIV/AIDS and HIV testing knowledge as information delivered orally by an HIV test counselor (a RA) among ED patients before rapid HIV testing [19]. After receiving the information from the video or counselor, participants assigned to the video and the orally-delivered information arms had similar mean scores for correct items (20.1 vs. 20.8) on the 26-item HIV/AIDS and HIV testing knowledge questionnaire from the previous pilot study. Also, the majority (94%) of participants in both study arms believed they were “well” or “very well informed” by the information they received [20]. Yet, English-speaking Latino patients had lower mean scores than non-Latinos, regardless of watching the video or having received information orally. This difference persisted even when controlling for years of education.

In 2010, we began a second study (R21NR011997) to adapt our English-language video, “Do you know about HIV and HIV testing?” into a 15-min Spanish-language video, “¿Qué sabe usted sobre el VIH y sobre las pruebas del VIH?” through a rigorous multi-step process. We conducted two rounds of cognitive-based assessments and pilot testing of the video and accompanying HIV/AIDS and HIV testing knowledge questionnaire among 18–64-year-old Latinos (*n* = 120) at three non-clinical community-based organizations (Chicago, Miami, and San Antonio), and three clinical sites of an ambulatory medical clinic (Providence), an ED (Los Angeles), and a department of health clinic (San Juan). In addition, we conducted similar assessments among 30 bilingual (English- and Spanish-speaking) Latino HIV test providers at the respective community-based organizations. We revised the video based on the results of the cognitive-based assessments and the pilot testing. During this process, we also adapted and tested an accompanying 25-item questionnaire designed to assess HIV/AIDS and HIV testing knowledge applicable to the goals of providing CDC-recommended HIV/AIDS and HIV testing information at the time of HIV testing. We verified the utility of the questionnaire through pilot testing and cognitive-based assessments (Cronbach’s α = 0.80).

We employed the Spanish-language video and questionnaire in a randomized, controlled, non-inferiority trial at an ambulatory medical clinic, an ED, and a community-based organization in Providence evaluating the efficacy of the video vs. comparable orally-delivered information from an HIV test counselor (a RA) in regards to short-term HIV/AIDS and HIV testing knowledge [21]. We enrolled 18–64-year-old primarily Spanish-speakers (*n* = 150) in the trial. Randomization was stratified by health literacy level (lower vs. higher) as measured by the Short Assessment of Health Literacy-Spanish and English (SAHL-S&E), which has strong psychometric properties in assessing health literacy [22]. After the video or orally-delivered information, HIV/AIDS and HIV testing knowledge applicable to CDC recommendations was assessed through audio-computer self-interviews (ACASI) using our previously developed 25-item questionnaire. Of the 150 participants, 39% met criteria for lower health literacy. For all participants, the mean scores for correct items on the questionnaire for the video (20.4; 95% CI: 19.5~ 21.3) and orally-delivered information (20.6; 95% CI: 19.7~ 21.5) groups (Δ = − 0.15; 95% CI: -1.4~ 1.1) were similar, satisfying the a priori non-inferiority criterion. Of those who watched the video, 93% stated that they were well or very well informed, and of those who received information orally, 96% stated that they were well or very well informed. Mean knowledge scores among lower health literacy participants were similar (18.3 [video] vs. 19.6 [in-person]; *p* < 0.30). In multivariable linear regression analyses, lower mean knowledge scores were related only to health literacy level (β − 2.4; 95% CI: -3.8, − 1.1), and not demographic characteristics (age, gender, race, insurance status, years of formal education, nativity, US acculturation), study arm (video or in-person information), study location (ambulatory medicine clinic, ED, or community-based organization) or prior HIV testing.

Following the trial, we made minor improvements to the video based on the results of the study (e.g., clarifying areas for which study participants scored less well). We also translated the Spanish version into English (“What do you know about HIV and HIV testing?”). Afterwards, we conducted a brief pilot study at the Rhode Island Hospital ED verifying short-term improvement in HIV/AIDS and HIV testing knowledge as measured by our 25-item HIV/AIDS and HIV testing knowledge questionnaire. Using a within-subjects comparison (i.e., pre- vs. post-vide0), short-term HIV/AIDS and HIV testing knowledge improved for both English and Spanish speakers (English: 22.8 vs. 17.9, Δ 4.89; *p* < 0.01; Spanish: 19.9 vs. 16.1, Δ 3.73; *p* < 0.001). Yet, overall testing knowledge was lower and improvement in knowledge was less among Spanish- than English-speakers.

Despite the data supporting the use of videos for delivering HIV/AIDS and HIV testing information in EDs, this communication medium has multiple disadvantages. Videos require electronic equipment; can be time consuming to watch; are more expensive (relative to brochures) to produce, update, and adapt for multiple cultures and languages; and might be “information overload” and off-putting for some patients, particularly those with higher health literacy and repeat HIV testers.

In contrast to videos, pictorial brochures have fewer disadvantages and are a promising alternative to videos. Similar to videos, pictorial brochures combine text and graphics to help illustrate concepts and might be better than text-only brochures for lower health and general literacy persons [23]. Pictorial brochures share the advantage of text-only brochures in ease of delivery, no training required to provide them, ability to be mass produced in multiple languages, and lower expense. However, we know of no published study evaluating pictorial brochures for delivering HIV/AIDS and HIV testing information, or studies comparing pictorial brochures to videos on this topic.

In summary, there currently exists a state of equipoise regarding optimal HIV/AIDS and HIV testing information delivery modes in EDs in light of CDC’s recommendations: (1) videos have been shown to be as efficacious as orally-delivered information; (2) informational brochures have not been adequately studied, although they are currently recommended; and (3) there have been no published studies evaluating pictorial brochures for HIV testing.

Although it is reasonable to assume that delivery of HIV/AIDS and HIV testing knowledge should be tailored to meet the needs of the estimated 15–40% of ED patients with lower health literacy [24–27], there is no published research on this topic. In particular, we do not know if pictorial brochures and videos are equivalently efficacious modes of delivering HIV/AIDS and HIV testing information, or if one mode is better than the other and should be preferred. Also unknown is how delivery mode affects retention of HIV/AIDS and HIV testing knowledge over time. Prior research indicates that intensive interventions [28–32] lead to retained HIV/AIDS and HIV testing knowledge. However, there have been no studies evaluating retention of knowledge from less intensive—yet much more likely to be used—modes of information delivery (e.g., brochures, videos*)*. Because previously studied intensive interventions (e.g., multiple skill-building sessions, peer counseling) are impractical in the ED setting, shorter, easier-to-deliver, yet efficacious modes are needed. In addition, it is important for maintain the operational efficiency of EDs to know if, when, how, and for whom information needs to be provided for HIV re-testing.

Information is believed to be necessary but not sufficient as a stand-alone component of HIV-related behavior change [33]. Although the videos we created address HIV- and HIV testing-related motivation and behavioral skills commensurate with the Information-Motivation-Behavioral Skills (IMB) model [34, 35], their impact on HIV test seeking behavior, utilization/acceptance of repeat HIV testing, and engaging in HIV risk-taking behaviors remains unknown. Furthermore, no studies have examined HIV re-testing after ED-based testing, and few non-ED studies have investigated factors associated with acceptance of HIV re-testing [36–39]. Understanding reported reasons for accepting or declining HIV re-testing is important for guiding future HIV re-testing interventions and approaches as well as addressing testing barriers. If HIV/AIDS and HIV testing information, and more specifically how it is delivered (pictorial brochure vs. video, according to health literacy level and language), impacts HIV re-testing behaviors, there would be better justification for providing this information and more attention on how (and to whom) it should be delivered.

If HIV/AIDS and HIV testing information delivery in EDs must be matched to patient health literacy, efficient and accurate ways to identify lower health literacy ED patients also need to be identified. Identifying adult ED patients with lower health literacy remains a challenge partly due to two limitations. First, there is limited research demonstrating the accuracy of extant screening instruments in EDs. Second, there are challenges in applying the screening instruments that have been demonstrated to be valid and reliable in identifying lower health literacy ED patients. The ideal health literacy instrument will be reliable and valid, brief, and easy-to-administer at the point of need in EDs.

A set of three questions that might fit these qualifications previously was derived from the Short Test of Functional Health Literacy in Adults (S-TOFHLA)), a longer, well-established screening device. These three questions address patient self-assessed health literacy skills according to three aspects: (1) need for help reading, (2) confidence with understanding, and (3) problems with reading medical forms [40–43]. Research conducted in hospital and outpatient settings assessing the utility of these three questions is promising. However, they have not been tested in ED populations, which can have greater diversity of demographic characteristics. Further, they only have been examined among English speakers, which further reduces their use in US EDs. In contrast, the SAHL-S&E [22] can distinguish between English- as well as Spanish-speakers with higher or lower health literacy. However, the SAHL-S&E comprises 18 questions, requires equipment (flash cards, data recorder, calculations), and must be administered by trained staff. This instrument is useful for research, but more difficult to use in routine ED practice. A short, easy-to-administer efficacious health literacy screening instrument could be used by ED staff to identify patients needing a particular mode of information delivery (e.g., video, pictorial brochure) to improve and retain HIV/AIDS and HIV testing knowledge.

### The current research

In our ongoing study (“Facilitating HIV/AIDS and HIV Testing Literacy for Emergency Department Patients”), we are conducting a multi-site, randomized, controlled, longitudinal trial with 600 English- and 600 Spanish-speaking 18–64-year-old ED patients. Our sample will be stratified by language (English or Spanish) and by health literacy level (lower vs. higher) assessed with the SAHL-S&E [22]. Participants will be randomly assigned within health literacy level to receive HIV/AIDS and HIV testing information by video or a pictorial brochure.

The video and pictorial brochure are designed to increase HIV testing knowledge, motivation, and behavioral skills and influence HIV re-testing behaviors. We will assess short-term (in ED) improvement in HIV/AIDS and HIV testing knowledge [21] and its longer-term retention over 12 months. At 12-months post-enrollment, we will offer participants the opportunity to be retested for HIV and we will measure differential testing uptake as a function of information delivery mode, language, and health literacy level. In addition, using the IMB model [34, 35] as a heuristic framework, we will examine IMB model study-relevant constructs and their interrelationships, their impact on HIV re-testing behavior, and moderation of information delivery mode, language and health literacy.

The innovative design of this study will permit us to determine, with adequate power, if pictorial brochures and videos are equivalently efficacious in improving and maintaining retention of HIV/AIDS and HIV testing knowledge, regardless of health literacy level and language preference. It will also allow us to determine if one mode of information delivery is superior or inferior, and under what circumstances (e.g., health literacy level and language) they are superior or inferior. The unique design will also permit a determination if, and for whom, HIV/AIDS and HIV testing information should be tailored. It also allows assessment of the impact of information delivery mode, language, and health literacy on acceptance of HIV re-testing. The findings will inform EDs whether information delivery modes should be selected for patients by language and/or health literacy level, or if either mode is preferred. The results will also tell EDs if, when, how, and for whom information should be provided for those re-testing for HIV within 1 year.

### Primary aims: HIV/AIDS and HIV testing knowledge acquisition and retention (information)

Determine how (Aim 1) short-term improvement and (Aim 2) longer-term retention of HIV/AIDS and HIV testing knowledge varies by information delivery mode (pictorial brochure or video), language (English or Spanish) and health literacy level (lower or higher) per four alternative hypotheses (HAs) (also provided are examples of outcomes of each HA):

- HA1 (3-way interaction of information delivery mode, language, and health literacy): e.g., improvement and/or retention of knowledge will be greatest among participants in the video arm who are English speakers and have higher health literacy, and will be least among those in the pictorial brochure arm who are Spanish speakers and have lower health literacy
- HA2 (2-way interaction of information delivery mode with language or health literacy): e.g., improvement and/or retention of knowledge will be greatest among participants in the video arm who are English speakers or higher health literacy patients, and will be least among those in the pictorial brochure arm who are Spanish speakers or lower health literacy patients
- HA3 (no interaction with information delivery mode): e.g., improvement and/or retention of knowledge will be greater in the video arm than the pictorial brochure arm regardless of language and health literacy level
- HA4 (no difference): improvement and/or retention of knowledge will be the same regardless of information delivery mode, language, and health literacy level (i.e., either a pictorial brochure or a video is useful for all patients)

### Secondary aims: HIV testing motivation, behavioral skills and behaviors

In addition to the information construct from the IMB model assessed in the primary aims, we will examine the IMB model constructs of motivation and behavioral skills, their interrelationships, their impact on HIV re-testing, and the moderating influence of information delivery mode, language and health literacy level on:

- Motivation: Improved/increased (1) attitudes toward need for HIV testing for oneself, (2) beliefs regarding the value of HIV testing, and (3) self-perceived risk for having an HIV infection;
- Behavioral skills: Increased skills and perceived self-efficacy on (1) initiating/seeking HIV testing, (2) interpretation and response to HIV test results, and (3) assessment of HIV risk and need for repeat testing;
- Behavior: Greater (1) HIV testing uptake 12-months post-enrollment when offered in the study, (2) HIV testing not part of the study during the study period, and (3) HIV testing utilization 12-months post- vs. pre-enrollment

#### Exploratory aims

To help apply the findings from our trial to ED practice, we will (1) explore brief, easy-to-use, single-question methods [40–43] that ED non-research staff could use to identify lower health literacy patients who would benefit more from information delivered via video or a pictorial brochure. We will also (2) assess participant satisfaction with their assigned information delivery mode and (3) their self-reported changes in HIV risk-taking behavior over the one-year follow-up period.

## Methods/design

### Theoretical framework of study

**Fig. 1:**
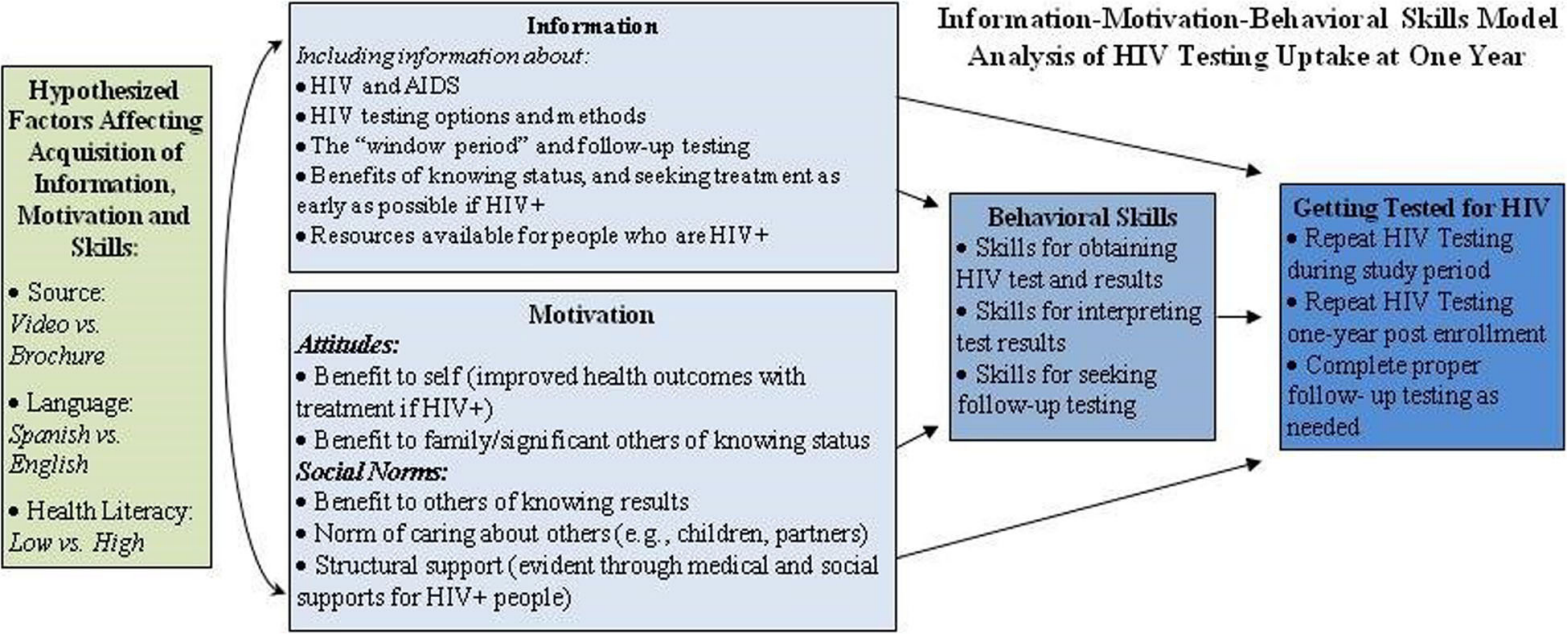
Information-Motivation-Behavioral Skills model as a theoretical framework for the trial

The study uses the IMB model (Fig. 1) to guide the selection of constructs and their measurement [34, 35]. Increase in and retention of information (knowledge) is the primary outcome, given that the chief purpose of the video and pictorial brochure is to provide HIV/AIDS and HIV testing information. However, the video and pictorial brochure also aim to increase motivation and improve behavioral skills related to HIV testing. Hence, changes in motivation and behavioral skills also will be measured. Given that HIV testing is a recurring event, especially for those at risk, we will measure the impact of the video vs. pictorial brochure on behaviors, chiefly acceptance of repeat HIV testing 12-months post-enrollment, as well as repeat testing during the study period and change in testing uptake at 12-months post enrollment. The interrelationships among knowledge, motivation, and behavioral skills, and their impact on testing behavior will be assessed. We also will examine the moderating influence of delivery mode, language, and health literacy level.

### Study sites and recruitment feasibility

**Fig. 2:**
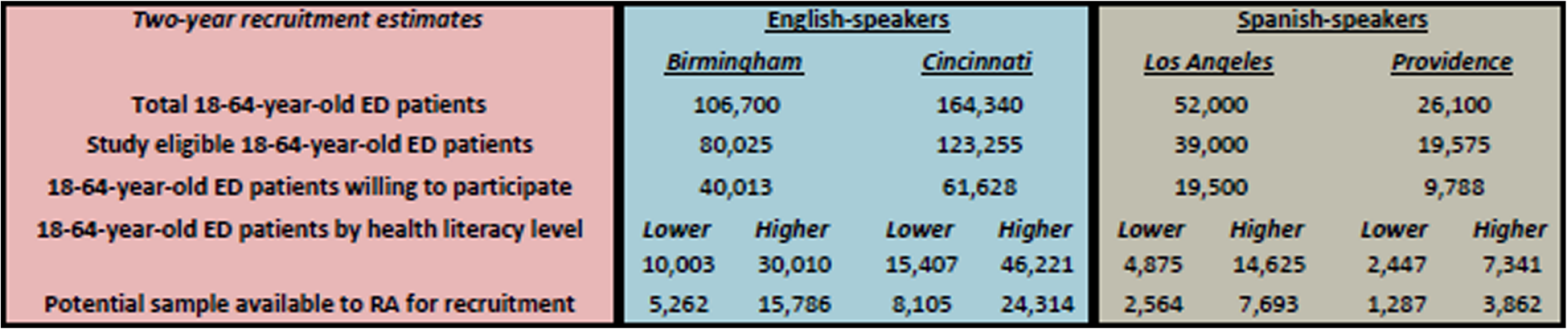
Participant recruitment feasibility estimates by study site, language, and health literacy level

We will conduct the study at four EDs (Birmingham: University of Alabama at Birmingham [UAB] Hospital; Cincinnati: University of Cincinnati Medical Center; Los Angeles: Olive View/University of California Los Angeles [UCLA] Medical Center; and Providence: Rhode Island Hospital) (Fig. 2, Table 1). The four study sites were selected because of their experience in conducting HIV testing research in their EDs, geographic and demographic diversity (permits sub-analyses by demographic characteristics), heterogeneity of HIV prevalence in the referring community, research infrastructure, and high ED patient volumes (enables easier recruitment). At two sites (Los Angeles and Providence), 38 and 15%, respectively, of ED patients only speak Spanish. Accordingly, we plan to recruit Spanish speakers at our Los Angeles and Providence sites, and English speakers at our Birmingham and Cincinnati sites.

**Table 1:** Descriptions of study sites

Location	Annual ED patient volume	ED patient demography	HIV prevalence
UAB Hospital ED	55,000 visits	• 54% black, 42% white, 3% Latino	• 0.4% in Jefferson County
Birmingham, AL	18–64-year-olds/year	• 53% female, 47% male	• 0.3% seroprevalence among those tested in ED
UCMC ED	83,000 visits	• 51% black, 44% white, 1% Latino	• 0.15% in Cincinnati metro area
Cincinnati, OH	18–64-year-olds/year	• 48% female, 42% male	• 0.4% seroprevalence among those tested in ED
Olive View-UCLA	63,000	• 5% black, 20% white, 70% Latino	• 0.38% in Los Angeles
Medical Center ED	18–64-year-olds/year	• 51% female, 49% male	• 4.4% seroprevalence among those tested in ED
Los Angeles, CA		• 75% of Latinos primarily speak Spanish	
Rhode Island Hospital ED	87,000 visits	• 10% black, 60% white, 30% Latino	• 0.2% in Providence metro area
Providence, RI	18–64-year-olds/year	• 48% female, 52% male	• 0.01% seroprevalence among those tested in ED
		• 50% of Latinos primarily speak Spanish	

We will recruit a total of 1200 patients (600 English-speakers and 600 Spanish-speakers) over a two-year period. The RA at each ED will recruit 300 ED patients over four shifts/week for 48 weeks/year for 2 years (384 shifts total/RA), and devote one shift/week to participant follow-up, training, and administrative duties. To reach recruitment goals, each RA must recruit at least 0.78 patients/shift. In comparison, we recruited 1.25 Spanish-speakers/shift in our previous R21 study (R21NR011997). Our recruitment estimates are conservative and assume that 25% of ED patients are study ineligible (see study inclusion criteria), 50% would decline participation, and only 25% would be classified as lower health literacy. As shown in Fig. 2, ample participants are available for recruitment at each ED per these assumptions.

### Study materials and instruments

#### HIV/AIDS and HIV testing video

“What do you know about HIV and HIV testing?” and its equivalent Spanish-language version “¿Qué sabe usted sobre el VIH y sobre las pruebas del VIH?” [21] is a 15-min animated and live-action video that contains CDC-recommended information about HIV/AIDS and HIV testing [2], as well as additional information about acute HIV infection and current methods of HIV testing (rapid and conventional; oral, fingerstick and phlebotomy sampling; and antibody, antigen, and ribonucleic acid testing) without mention of any type of testing product. The video may be downloaded or viewed without cost at http://biomed.brown.edu/hiv-testing-video/. The content of the video is grounded in the IMB model [34, 35] with a primary emphasis on improving knowledge about HIV/AIDS and HIV testing, while also increasing motivation for testing and improving behavioral skills regarding HIV testing. (See Additional file 1 for details of content according to IMB model components). The voice-over narrated video follows two protagonists (male and female; purposely racially/ethnically ambiguous and not named to appeal to a wider audience and avoid social labels) as they receive information about HIV/AIDS and HIV testing and proceed through the HIV testing process. Animation, graphics, images, still shots, text, and live-action segments emphasize the topics presented. The Fernandez-Huerta Readability Score [44] for the Spanish-language version of the script for the video is 85, which indicates an “easy” level of reading difficulty. The final English-language version of the video script has a Flesch reading ease of 72.9, indicating a low reading ability level (appropriate for 11-year-olds).

#### HIV/AIDS and HIV testing pictorial brochure

The HIV/AIDS and HIV testing pictorial brochure is a compact printed version of the video, “What do you know about HIV and HIV testing?” and its equivalent Spanish-language version, “¿Qué sabe usted sobre el VIH y sobre las pruebas del VIH?” [21] It contains identical information as the video, except there is no voice-over narration, music, animation, or live-action segments. Instead, graphics, images, and still shots of selected components of the animated and live-action segments are depicted. The Fernandez-Huerta Readability Score [44] and the Flesch reading ease are identical to the video. English- and Spanish-language copies of the text of the video and pictorial brochure are in the Additional file 1.

### Study instruments (see Additional file 1 for English-language copies)

Table 2 depicts the study instruments that will be used in the study.

**Table 2:** Study instruments description and administration

Instrument	Description	Administration
Screening, eligibility and enrollment questionnaire	• Karliner, et al. Spanish language proficiency & preference [54]	• Baseline
	• Ballard and Tighe Idea Proficiency Test II	• Administered by RA
	• Demographic characteristics & nativity	• < 5 min
	• US acculturation (for Latinos) per the SASH [63]	
	• HIV testing history & HIV-related exclusion screen	
	• Willingness to undergo rapid HIV testing	
	• Adapted from our prior research [19, 21, 45–51]	
Short Assessment of Health Literacy-Spanish & English (SAHL-S&E [22])	• Measures health literacy level	• Baseline
	• Score of ≤14 indicates lower health literacy	• Administered by RA
	• *SAHL-S* is highly correlated with *TOFHLA* (*r* = 0.62), & *SAHL-E* is highly correlated with *REALM* (*r* = 0.94) & *TOFHLA* (*r* = 0.68)	• 3–5 min
	• *SAHL-S&E* reliability is 0.80 & 0.89, respectively	
3 single-item screening tests for health literacy [40–43]	• Derived from the S-TOHFLA	• Baseline
	• AUCs for 3 questions vs. S-TOHFLA (0.66–0.74) vs. REALM (0.72–0.84)	• Administered by RA
		• < 1 min for each question
HIV testing motivation and behavioral skills questionnaire (Behavioral Skills and motivation)	• Measures **Motivation:** (1) attitudes toward need for HIV testing for oneself, (2) beliefs regarding the value of HIV testing, and (3) self-perceived risk for having an HIV infection; and **Behavioral skills:** skills and self-efficacy on (1) initiation/seeking of HIV testing, (2) interpretation and response to HIV test results, and (3) assessment of HIV risk and need for repeat testing	• Baseline (pre- and post-information by video or pictorial brochure)
	• Adapted from our prior research [47, 49, 51] and other author recommendations [74]	• Self-administered by telephone
		• < 2 min
HIV/AIDS and HIV testing information delivery mode preferences and satisfaction questionnaire	• Measures preferences and satisfaction with pictorial brochure or video	• Baseline (pre- and post-information by video or pictorial brochure)
	• Adapted from our prior research [20]	• Self-administered by telephone
	• 1 Pre- and post-information question on preferences with delivery mode	• 1 min
	• 1 post-information question on satisfaction with delivery mode information	
HIV/AIDS and HIV testing knowledge questionnaire [21] (Information)	• Measures HIV/AIDS and HIV testing knowledge applicable to CDC-recommended information at the time of testing	• Baseline (pre- and post-information by video or pictorial brochure)
	• Cronbach’s α = 0.80; *See A.1. Preliminary research for more details about development, testing, and utilization of this instrument*	• 3, 6, 9 and 12-month follow-up
	• 25 questions; yes/no/don’t know responses	• Self-administered by telephone
		• 5–10 min
HIV risk-taking questionnaire	• Measures number of partners and frequency of condom usage by gender and type of partner (main, casual and exchange partners) and injection-drug use	• Baseline
	• Based upon CDC National HIV Behavioral Surveillance System (NHBS)	• 3, 6, 9, and 12-month follow-up
	• Adapted from our prior research [47, 49, 53]	• Self-administered by telephone
		• 5–10 min
HIV test utilization questionnaire	• Queries participants about HIV testing in the follow-up period	• 3, 6, 9, and 12-month follow-up
	• Adapted from our prior research [47, 49, 51]	• Self-administered by telephone
		• < 2 min
Repeat rapid HIV testing acceptance questionnaire	• Measures participant acceptance of offer of repeat rapid HIV testing at 1 year post-enrollment and reasons for acceptance/decline	• 12-month follow-up
	• Adapted from our prior research [47] and research by other authors [49, 51, 75–80]	• Self-administered by telephone
		• < 2 min

### Study preparation (months 1–6): RA hiring and training, study preparation, and brief pilot study

#### RA hiring and training (months 1–5)

We will work with the study site investigators to hire and train RAs at each study site. The project manager, principal investigator (PI), co-investigators, and site investigators will conduct training of the RAs in person through site visits and video conferences. We will ensure that the RAs are adept in screening and enrolling participants and executing the protocol of the trial. As we have done for our prior studies [19–21, 45–51], the RAs also will undergo their respective state-sponsored HIV counseling and testing training, and instruction on rapid HIV testing with the PI and the rapid HIV test manufacturers.

#### Study instrument preparation (months 1–2)

We will prepare our study instruments for each trial site and for the follow-up components. The baseline English and Spanish versions of the enrollment and eligibility screening and health literacy questionnaires will be loaded onto tablet computers for the QDS™ software data collection system (NOVA Research, Bethesda, MD), which we have used in our previous studies [17, 19–21, 45–50, 52, 53]. We also will load our video, “What do you know about HIV and HIV testing?” (English- and Spanish- versions) [21], onto the tablet PCs. Finally, we will prepare the rapid HIV testing data collection and quality assurance forms and protocols we used for our prior studies for each study site [19–21, 45, 47–49, 51, 53].

We considered a variety of methods of administering the remaining questionnaires for the baseline and consecutive three-month follow-up periods. We need a system that is convenient for participants; can be accessed from virtually any location in the US; is available 24 h/day, 7 days/week; is easy-to-use for participants; can deliver the questions in English or Spanish; and is responsive to those with lower literacy skills. Considering these needs, we will use the Database Systems Corporation automated telephone key-pad response system for delivering the questionnaires at baseline and for each follow-up. Participants will be given a toll-free telephone number to call and complete the questionnaires. Participants will use this system at study entry in the ED with the on-site assistance of the RA. In so doing, we will verify that participants know how to use the system. Those participants who are having difficulty with using the system during follow-up will have the option to provide their responses with the assistance of the RA through a telephone call.

#### Brief pilot study (month 6)

We will conduct a brief pilot study to finalize the study protocol. The pilot study will serve to further train the RAs, enable us to “trouble-shoot” the study methods, gather some pilot data to review and ensure accuracy and completeness of the study data, and rectify any problems. For the pilot study, each RA will enroll five participants. Study inclusion criteria and protocol will be the same as for the randomized, controlled trial (provided below).

### Randomized, controlled trial (recruiting months 7–42; follow-up ends month 54)

#### Study inclusion criteria

- 18–64-years-old (per CDC HIV screening recommendations [1])
- Speak English or Spanish
- Negative baseline rapid HIV test (described below)
- Not enrolled in a conflicting HIV study (described below)
- Can provide informed consent and can participate
- Plan to remain in the US for 12 months

#### Enrollment (months 7–42)

The RAs will approach a random sample of ED patients who might be eligible for the study. The random selection process entails a computer-based selection of ED patients to approach, as we have done in our previous studies [19, 20, 45, 47–51]. In brief, this process involves creating lists of ED patient rooms, entering them into the computer-based random selection program (www.random.org), and generating a list of the rooms in random order in advance of each data collection shift. The RA will review the electronic ED medical records of patients according to these lists and determine which patients are potentially study eligible.

**Fig. 3:**
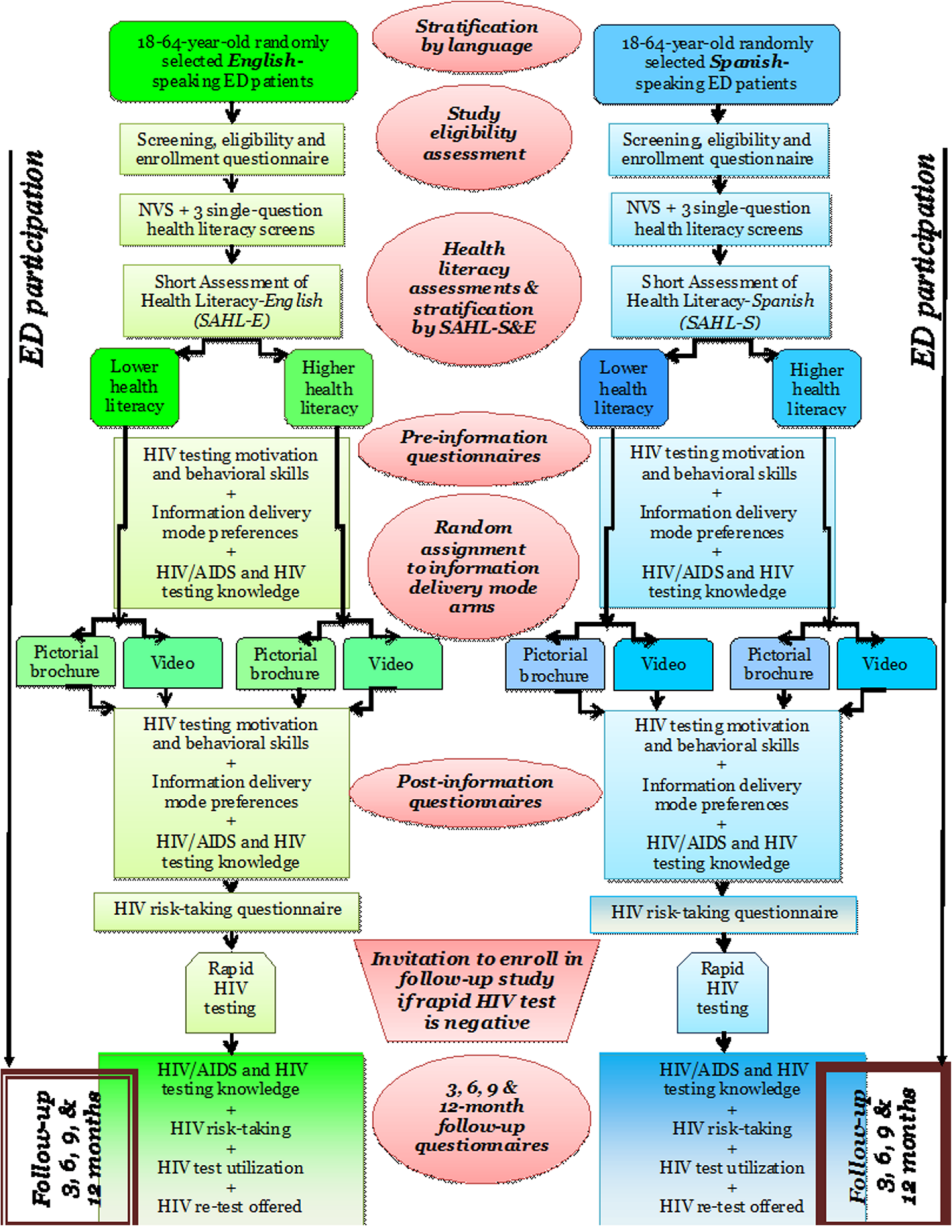
Trial protocol flow diagram

The RAs will approach ED patients who appear to be eligible and conduct a further in-person screen of their eligibility. Study screening enrollment and participation will be stratified by language (English or Spanish) (Fig. 3). Ability to read and comprehend English and Spanish at least at a 6th grade level will be verified using the Ballard and Tighe Idea Proficiency Test II Form E for English language and the fourth edition for Spanish language (Ballard and Tighe Publishers, Brea, CA). Patients who self-identify as primarily Spanish speakers also will complete the two-question screener by Karliner et al. [54] to confirm preference for Spanish-speaking in medical settings. Afterwards, they will complete the Spanish-language version of the questionnaires and receive the applicable study materials. Patients who decline or abort screening or study participation at any time will be asked to provide their reasons, which will be recorded in the study database. If the reason can be overcome (e.g., waiting for a radiograph), the RAs will accommodate the request, and will proceed with screening or participation.

For those who agree to be screened (verbal consent will be obtained), the RAs will administer the screening, eligibility and enrollment questionnaire (*See* Additional file 1). The screening questionnaire consists of questions about demographic characteristics and HIV testing history. Patients who self-identify as HIV infected or are enrolled in conflicting HIV studies (e.g., HIV vaccine, HIV pre-exposure prophylaxis [PrEP], or a study that measures HIV testing over time or similar outcomes as this study) will not be eligible and will be thanked for their time. Next, the RA will administer the three health literacy questions [40–43]. The RA then will administer the SAHL-S&E [22] to stratify participants by health literacy level (lower or higher). During the course of the latter portion of the study if a participant’s health literacy level exceeds the quota for the study site, he/she also will not be eligible for participation.

#### Pre-information delivery questionnaires

Patients agreeing to participate will complete the pre-information delivery questionnaires with the automated telephone key-pad response system using the RA’s cellular phone. These instruments are the HIV risk self-perception and value of HIV testing, HIV/AIDS and HIV testing information delivery mode preferences, and the HIV/AIDS and HIV testing knowledge questionnaires (See Additional file 1). Verbal consent will be obtained for this portion of the study.

#### Information delivery and post-information delivery questionnaires

We will randomly assign patients in a 1:1 ratio into information delivery mode Arm 1 or Arm 2. As noted previously, the two arms will be stratified within language (English or Spanish) and health literacy level [22] (lower vs. higher) (*n* = 150/stratum). We will use block randomization techniques to ensure that there are equal numbers in each arm per study site and stratum using a centralized, random assignment computerized system. Randomization sequences by language and health literacy level will be posted on a secure website that RAs across all four sites will consult when assigning participants to study arms. The advantage of this approach is that we can monitor enrollment and balance within study arms and across study sites easily, address concerns as they arise, and permit efficient use of study resources. The two groups will differ by the following:

- Arm (1): HIV/AIDS and HIV testing video: Participants in Arm 1 will watch the video, “What do you know about HIV and HIV testing?” (in English or Spanish) on a tablet computer in the ED while the RA waits. They will listen to the audio components using disposable headphones.
- Arm (2): HIV/AIDS and HIV testing pictorial brochure: Participants in Arm 2 will be provided with a copy of our HIV/AIDS and HIV testing pictorial brochure (in English or Spanish) to review in the ED. Arm 2 participants will read the brochure while the RA waits. The RAs will not provide clarifications or assistance with the information in the pictorial brochure.

After watching the video or reviewing the pictorial brochure, all patients will complete the post-information questionnaires using the automated telephone key-pad response system (the HIV risk self-perception and value of HIV testing, HIV/AIDS and HIV testing information delivery mode preferences and satisfaction, the HIV/AIDS and HIV testing knowledge, and the HIV risk-taking questionnaires). Patients will receive a re-chargeable gift card for participating.

#### Rapid HIV testing and assurance of linkage-to-care

When approaching ED patients about the study, the RAs will inform them that for the study they will have an HIV test after completing the post-information questionnaires. They also will be informed that their test result must be negative to be enrolled in the longitudinal, follow-up portion of the study. The negative HIV test is required because one of the aims of this study is to assess retention of the information provided via pictorial brochure or video, which is pertinent only for those with a negative HIV test. Those who are HIV infected would not require repeat testing or receipt of this information.

After each patient has completed the initial questionnaires and received HIV/AIDS and HIV testing information from the pictorial brochure or video, the RAs will briefly describe the test procedures. The RAs will record the reasons for accepting or declining HIV testing (*See* Additional file 1). Those who decline testing will be provided with a brochure about HIV/AIDS and HIV testing that contains a list of places where they can be tested in their area. Those who agree to testing will sign a consent form which specifies that: (1) the participant will undergo HIV testing and the results of their test will be included in the study database; (2) participation in the longitudinal, follow-up portion of the study is contingent upon a negative HIV test; (3) the RA will obtain contact information for the participant to ensure attendance at the follow-up appointment and receipt of the final confirmatory test results (for those with a preliminary positive test); (4) the RA will initiate confirmatory testing for HIV for those who test preliminary positive for HIV; and (5) the RA will assist them in adhering to their follow-up visit. The consent form will also outline the follow-up procedures and participant responsibilities for the study.

The type of HIV testing conducted will vary by study site based on standard practice at each location. The Los Angeles and Providence sites will use fingerstick rapid HIV testing performed by the RA. The Birmingham and Cincinnati sites will use laboratory-based HIV testing. Participants will receive their test results during their ED study encounter. The RAs, as trained HIV test counselors, will provide post-test counseling and support for those with a preliminary positive HIV test result. The RA will coordinate with ED staff to obtain a phlebotomized sample for CD4+ count, Western blot, and viral load testing. We will adapt the testing, quality assurance, preliminary positive test, and assurance of linkage-to-care protocols from our prior studies, that include obtaining extensive contact information from the participant, providing multiple means of contacting study staff, written information detailing the appointments for follow-up, reminder telephone calls, and assistance with transportation to the follow-up appointment. The project manager, PI, and site investigators also will help to ensure linkage-to-care.

#### Follow-up assessments (months 10–54)

Potential participants meeting all study eligibility criteria (including a negative HIV test) will be invited to enroll in the longitudinal, randomized, controlled trial. Participants will complete the follow-up assessments via the automated telephone questionnaire system at 3, 6, 9 and 12 months post-enrollment. Prior to the due dates of each participant’s 3, 6, 9, and 12-month follow-up, the RAs will remind each participant by telephone, email, and letter that their follow-up is due, how to complete the follow-up, and who to contact for help. During the telephone assessments, participants will complete the HIV/AIDS and HIV testing knowledge, HIV risk-taking, and HIV testing utilization questionnaires. Participants who have difficulty with the telephone system can contact study staff and complete the assessments with their assistance. After completing each follow-up assessment, each participant’s gift card will be re-charged (in successively increasing amounts). Those who report to us that they no longer have their gift card will be sent a new one. Any participants who fail to complete the 3-month post-enrollment follow-up will be discontinued from the study. The recruitment quota will be adjusted to replace them as applicable at their respective study site.

#### Repeat rapid HIV testing

As a final step in the 12-month follow-up assessment, all participants will be offered the opportunity to be tested (again) for HIV. Study staff will call these participants and offer to send them a free rapid HIV “home self-test” (OraQuick™ In-home rapid HIV test [55, 56]) to be delivered to the address they select and that they perform themselves. They will be given the alternative of taking the test kit to their study recruitment site and have the RA perform the test for them. We will contact those who elected to undergo HIV testing monthly for up to 3 months to inquire about their use of the test and the test results. Those whose test is preliminary positive will undergo confirmatory testing at their study site.

#### Retention of study participants

RAs will obtain and verify each participant’s contact information (telephone numbers, email addresses, pager/text numbers, mailing and housing addresses, shelters, relatives/friends’ contacts) at enrollment. Within 1 week, we will call each participant, verify their contact information, send a welcome letter and email about the study participation requirements, and remind the participant about the expectations of the study. If a participant cannot be reached, or otherwise fails to perform the follow-up assessments, we will send a certified letter or a courier to their last known address. If needed, two RAs from each study site will go in person to the last known addresses of that participant (home, work, shelter, or relatives/friends’ contacts) to locate them. Two RAs will jointly perform these site visits to ensure their safety. The RAs will either obtain new contact information from the participant or their contacts, or provide the participant with a cellular phone immediately to complete the follow-up assessments.

#### Sample size considerations

**Fig. 4:**
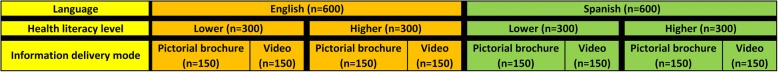
Sample size by study arm, language, and health literacy level

The study sample size is *n* = 1200, with equal size strata (*n* = 150) by language, health literacy level, and information delivery mode (Fig. 4). This sample size is based on a 2x2x2 full factorial design and will satisfy the requirements for analysis of the two primary aims and the four alternative hypotheses paired with each aim. The minimum sample size is calculated for a power of 0.80 and a two-sided *α = 0.05* level of significance (Table 3). In general, a larger sample size is needed for greater variation of scores within study arms and strata, lower within-person correlations of scores, and higher loss-to-follow-up. To maximize power, we chose conservative values based upon our prior studies. We increased the sample size by 20% to account for the heterogeneity in patients among the four study sites, and by a conservative additional 25% to account for possible attrition. Based upon other 12-month ED intervention follow-up studies [10], HIV/STD testing studies [57–59], and studies conducted at our own ED [60, 61], a 25% attrition rate at 12 months likely is a worst-case scenario. We expect that our experience and enhanced measures to encourage and facilitate follow-up will result in higher follow-up rates. As of publication of this manuscript, 1165 participants enrolled completed the three-month follow-up.

**Table 3:** Sample size rationale by primary aims

Primary Aims	Minimum sample by stratum	Total minimum sample	Rationale and assumptions for minimum sample sizes and final sample sizes for each primary aim
Aim 1. Short-term improvement in HIV/AIDS and HIV testing knowledge ** HA1**: 3-way interaction (mode*language*literacy level) ** HA2**: 2-way interaction (mode*language or literacy level) ** HA3**: no interaction (difference by mode only) ** HA4**: no difference	**125** This **minimum** sample size by stratum will yield a power of 0.80, 0.97 and 0.99 for testing HA1, HA2, HA3 versus HA4 (HA4 as the NULL) Final sample per stratum (information delivery mode, language, and health literacy level) is 150	**1000** The **final sample** after adding 20% to account for the heterogeneity in patients among study sites is **1200** = 1000*(1 + 20%)	• Based on our prior video studies [17, 19, 21], we assume that the scores on the 25-item questionnaire is a normal distribution with a standard deviation of ~ 3 • Before information delivery, mean scores of the two arms are assumed to be the same (due to random assignment) per stratum • Short-term within-person correlation is 0.7 or higher [21] • The pictorial brochure will improve knowledge by an average of 2 points. The video will improve the knowledge by an average of 3.5 points. The effect size is Δ = 3.5–2 = 1.5. (HA3) • 2-way intrxn (mode*language or literacy level) effect size is Δ = 1.5 + 0.75 = 2.25. (HA2), e.g. for those speaking English OR with high health literacy • 3-way intrxn (mode*language*literacy level) effect size is Δ = 1.5 + 0.75 + 0.75 = 3.0, e.g. for those speaking English AND having high literacy AND watching the video
Aim 2. Retention of HIV/AIDS and HIV testing knowledge at 3, 6, 9, and 12 months ** HA1**: 3-way interaction (mode*language*literacy level) ** HA2**: 2-way interaction (mode*language or literacy level) **HA3**: no interaction (difference by mode only) ** HA4**: no difference	**100** This **minimum** sample size by stratum will yield a power of 0.80, 0.97 and 0.99 for testing HA1, HA2, and HA3, versus HA4 (as the NULL)	**800** After adding 25% to account for loss-to-follow-up and 20% for heterogeneity among study sites, the **final sample** size is **1200** = 800*(1 + 25%)*(1 + 20%)	• Long-term within-person correlation is 0.4 or higher • The scores on the knowledge questionnaire in the pictorial brochure arm will degrade to the pre-information baseline level with an average drop of 2 points by 12 months. The score in the video information arm will degrade by an average drop of 1 point. So, compared to baseline, the difference (effect size) in long-term retention of information in the video arm as compared to the pictorial brochure arm is Δ = (3.5–1)-(2–2) = 2.5. • We will assume a similar effect size for 2- and 3-way interactions

### Study analysis (begins at end of recruitment month 43)

#### Enrollment summary and comparison of participants at baseline

We will report on participant enrollment and summarize the reasons for accepting or declining enrollment or drop-out from the study using the Consolidated Standards of Reporting Trials (CONSORT) approach [62]. We will summarize the demographic characteristics, HIV testing history, and health literacy level (measured by the SAHL-S&E [22] and brief instruments) using conventional statistics, such as means/standard deviations, medians/ranges, or counts/frequencies with corresponding 95% confidence intervals (CIs), as appropriate. For Latino participants, we will summarize nativity and US acculturation (measured by the SASH [63, 64]) in a similar manner. We will summarize these baseline characteristics for all participants and as stratified by informational delivery mode (pictorial brochure or video), language preference (English or Spanish), and health literacy level (lower or higher). Next, we will compare the baseline characteristics of study participants between the study arms using Pearson’s Χ^2^ or Fisher’s exact test for categorical variables and Student’s t-test for normally distributed or Wilcoxon’s rank-sum test for non-normally distributed continuous variables to assess the success of the randomization procedure. Any chance imbalances between the two arms will be documented and investigated, and will be adjusted for by including the imbalanced covariate as an independent variable in our subsequent regression analyses, or through a weighted analysis using a propensity score (propensity to be enrolled in the study with weights equal to the inverse of the propensity score) [65]. A two-tailed, *α = 0.05* significance level will be used for all analyses.

#### Primary aims analysis

For Aim 1, we expect that HIV/AIDS and HIV testing knowledge scores can be reasonably approximated by a normal distribution (Table 4). We will compare differences in pre- vs. post- changes in scores between information delivery modes by paired t-tests. If the data are not normally distributed, we will use signed-rank tests. All tests will be executed on an intention-to-treat basis (non-adherence to assigned information delivery mode likely is minimal). Comparisons will first be made for each stratum. MANOVA/ANCOVA will be used for combined samples where language and literacy level are used as stratifying (main) factors. To further account for baseline covariates (e.g., demographic characteristics), a generalized linear mixed-effect model will be fit to the data. The parameters of interest are the coefficients of the dummy variable indicator of study arm assignment and its interactions with language and health literacy level.

**Table 4:** Analyses of primary aims

Primary aims	Measurements	Analytic methods
Aim 1. Short-term improvement in HIV/AIDS and HIV testing knowledge **HA1**: 3-way interaction (mode*language*literacy level) **HA2**: 2-way interaction (mode*language or literacy level) **HA3**: no interaction (difference by mode only) **HA4**: no difference	HIV/AIDS and HIV testing knowledge questionnaire at baseline in ED at time of HIV testing, post- vs. pre-information delivery	• Paired t-tests • Signed-rank tests • MANOVA/ANCOVA • Generalized linear mixed-effects regression
Aim 2. Retention of HIV/AIDS and HIV testing knowledge at 3, 6, 9, and 12 months **HA1**: 3-way interaction (mode*language*literacy level) **HA2**: 2-way interaction (mode*language or literacy level) **HA3**: no interaction (difference by mode only) **HA4**: no difference	HIV/AIDS and HIV testing knowledge questionnaire at 3, 6, 9, and 12 months vs. baseline (post-information delivery)	• Paired t-test for each follow-up vs. baseline • Linear mixed-effects regression (assuming constant degradation over time) • Broken-stick linear mixed-effects regression (for non-constant degradation over time)

The generalized linear mixed-effect model will be structured as follows: Y^*^_ij_ *= α*_*j*_ *+ f(R*_*i*_*,LG*_*i*_*,LT*_*i*_*; β) +* μ_i_ *+ θX*_*i*_*,* where *j = 0,1* indicates pre- and post-information, Y^*^_ij_ is the outcome of subject *i*, R_i_ indicates the arm assignment, *α*_*j*_ is the intercept (*α*_*0*_ being the pre-information value for j = 0; and *α*_*1*_ the post-information value for j = 1), *β* is a vector that captures the main effects, and 2- and 3-way interactions among information delivery mode *(R),* language *(LG*), and literacy *(LT)*, *f* () is a linear predictor function, and *X*_*i*_ contains important or stratification covariates, with *θ*θ being their effects. For this unified approach, *Y*_ij_^*^ = *g*[E (*Y*_*ij*_)] where *g*() is an appropriate transformation of the outcome being analyzed. For example, for continuous outcomes, *g*() can be an identify link; for count or binary outcomes (as in the exploratory aims), *g*() can be a log or logit/probit transformation. We will assume that *u*_*i*_ *~ N(0, τ*^*2*^*)*, which is a random effect that captures the within-subject correlation of the repeated measurements. We will fit the models using the restricted maximum likelihood method, or generalized estimating equations (GEE) if non-identify links are used.

For Aim 2, we will first compare the baseline literacy scores at each of the follow-up visits to the baseline score (post-information delivery) using paired *t*-tests. If the retention of HIV/AIDS and HIV testing knowledge demonstrates a linear trend over time, we will fit a linear mixed-effect model to the combined data, in which independent variables will include time, information delivery mode, language and baseline health literacy level; their interactions; age, gender, etc. If the trend is not linear, we will consider fitting a broken-stick (piecewise linear) linear mixed-effect model. The parameters of interests are the coefficients of time, indicating follow-up assessments, and the interactions of time and study arm assignment, information delivery mode and language.

If a linear assumption is supported by data, the linear predictor in the above model will be expressed as *α*_*0*_ *+ β*_*1*_*[time] + β*_*2*_[time]*R_*i*_*[mode]*, where *β*_*1*_ and *β*_*2*_ capture HIV/AIDS and HIV testing knowledge retention rate over time and the difference in the rate between two delivery modes. We will further evaluate the 2- and 3-way interactions *β*_*2*_[time]*[language]*R_*i*_ *[mode]* and *β*_*2*_[time]*[health literacy level]*R_*i*_*[mode]* and possibly (if data allow) 4-way interactions *β*_*2*_[time]*[language]*[health literacy level]*R_*i*_*[mode]*, to investigate the interaction of information delivery mode with language and health literacy and whether one delivery model works better in certain sub-groups. Our analysis by time will permit us to determine if and when HIV/AIDS and HIV testing knowledge degrades, and if and when knowledge reaches baseline pre-information levels (i.e., before an intervention), which would suggest a need for repeat delivery of this information at subsequent HIV testing encounters within 12 months. We also will determine if degradation varies by information delivery mode, language, and health literacy level. We recognized that there is no established minimum standard for what level of HIV/AIDS and HIV testing knowledge is necessary or desired; however, we believe that it should be at least greater than pre-information knowledge levels, and will plan the analyses accordingly.

#### Secondary analyses of primary aims

Our a priori secondary analyses entail examining if there are sub-groups with differences in HIV/AIDS and HIV testing knowledge improvement or retention in the short- and longer-term. We will assess the impact of demographic characteristics, HIV testing history, and testing site for all participants, as well as nativity and US acculturation for Latino participants in these secondary analyses. For these secondary analyses, we will use the aforementioned generalized linear mixed-effect models to take into account the two-arm clinical trial design; language and health literacy level; the repeated measures, the longitudinal data that are collected at baseline and follow-up assessments; and the covariates of interest (e.g., demographic characteristics and HIV testing history). Our focus of analysis will be the main effects of the covariates of interest and their interaction effects with study arm, stratifying factors and time. For longer-term retention of knowledge, we also will examine the impact of repeated testing during the follow-up period.

#### Secondary aims and exploratory aim analyses

Our main secondary aim is assessing HIV re-testing behavior (Table 5). For our sample size (*n = 150/stratum*), we will have adequate power to detect a ≥ 15% absolute difference (Δ) in HIV testing behaviors (re-testing at 12 months, HIV testing outside of the study) by group within strata (information delivery mode, language and health literacy level) (assumes ≥75% follow-up, β = 0.2, α = 0.05). A ≥ 15%Δ is reasonable, per other HIV testing research [16, 66, 67]. Per the IMB model, we also will investigate differential changes in short-term (in ED) motivation and behavioral skills by information delivery mode, language, and health literacy level. Further, we will (1) assess if increase and retention of knowledge, motivation, and behavioral skills impacts behaviors (HIV testing); (2) examine the interrelationships of model components (e.g., path coefficients [34]); and (3) assess the moderating influence of information delivery mode, health literacy level and language on motivation, behavioral skills and behavior change. Mediation/moderation models using structural equation modeling and other recommended approaches will be used [68–73]. We also will examine other factors potentially associated with HIV re-testing, such as demographic characteristics, study site, and changes in HIV risk-taking behavior over time.

**Table 5:** Analyses of secondary and exploratory aims

***Secondary aims***	***Measurements***	***Analytic methods***
**Motivation** (1) attitudes toward need for HIV testing for oneself, (2) beliefs regarding the value of HIV testing, and (3) self-perceived risk for having an HIV infection	HIV testing motivation and behavioral skills questionnaire; post- vs. pre-information delivery	• Paired t-tests or signed rank tests • Generalized linear mixed-effects regression
**Behavioral skills** (1) initiating or seeking of HIV testing, (2) interpretation and response to HIV test results, and (3) assessment of HIV risk and need for repeat testing	HIV testing motivation and behavioral skills questionnaire; post- vs. pre-information delivery	• Paired t-tests or signed rank tests • Generalized linear mixed-effects regression
**Behaviors** (1) testing uptake one-year post-enrollment when offered in the study, (2) testing utilization during the study period, and (3) change in testing utilization one-year pre- vs. post-enrollment	(1) Repeat rapid HIV testing acceptance questionnaire (2) HIV test utilization questionnaire (3) HIV testing history	• McNemar’s tests • Cochran-Mantel-Haenszel’s test • Generalized linear mixed-effects model
***Exploratory aims***	***Measurements***	***Analytic methods***
Utility of brief health literacy instruments	NVS and 3 single-item screens of health literacy level vs. SAHL-S&E and HIV/AIDS and HIV testing knowledge questionnaire	• Pearson/Spearman correlation • Cohen’s Kappa for agreement tests • ROC analyses • Test performance parameters
Information delivery mode preferences and satisfaction	HIV/AIDS and HIV testing information delivery mode preferences and satisfaction questionnaire; post- vs. pre-information delivery	• Paired t-tests or signed rank tests • Generalized linear mixed-effects regression
HIV risk-taking behaviors	HIV risk-taking questionnaire	• McNemar’s tests • Cochran-Mantel-Haenszel’s test • Generalized linear mixed-effects model

For the exploratory aims, we will investigate whether the three brief health literacy screening questions are useful in the ED to identify patients with lower health and lower HIV/AIDS and HIV testing knowledge. Their utility and accuracy to screen for patients with lower health literacy as a substitute for the SAHL-S&E (which is a research instrument not practical for typical ED use), compared to this “gold standard” instrument will be assessed. Also, their ability to identify patients with lower HIV/AIDS and HIV testing knowledge will be assessed using ROC-type analysis, and quantified by test performance parameters (e.g. C-statistic). We also will compare participant satisfaction with the information delivery mode they received and the potential influence of information delivery mode (and differences by language and health literacy level) on changes in HIV risk-taking behavior over the study period and pre- vs. post-enrollment.

## Discussion

This research project ultimately aims to guide best practices for HIV testing in the emergency medicine setting. Its objective is to learn how HIV/AIDS and HIV testing should be delivered (video or pictorial brochure) and whether health literacy and language spoken influences improvement in knowledge, motivation and behavioral skills around testing, HIV risk-taking behaviors, and re-testing for HIV. The project intends to inform EDs if the delivery of HIV/AIDS and HIV testing information should vary by health literacy and language spoken, and if resources should be invested to provide this information at every testing encounter. In addition, by examining potential easier to administer health literacy screening tests, EDs might be able to identify quickly those patients who might need more assistance. We look forward to obtaining the data and analyzing it to address the aims of the project.

## Additional file

Additional file 1: Study instruments and video/pictorial brochure elements and script. (DOC 393 kb)

## Abbreviations

ACASI: Audio computer-assisted self-interview
ANCOVA: Analysis of covariance
CDC: Centers for Disease Control and Prevention
CONSORT: Consolidated Standards of Reporting Trials
ED: Emergency department
HAs: Alternative hypotheses
IMB: Information-Motivation-Behavioral Skills model
MANOVA: Multivariate analysis of variance
PI: Principal investigator
PrEP: Pre-exposure prophylaxis
RA: Research assistant
SAHL-S&E: Short Assessment of Health Literacy-Spanish & English
STD: Sexually transmitted diseases
S-TOFHLA: Short Test of Functional Health Literacy in Adults
UAB: University of Alabama at Birmingham
UCLA: University of California, Los Angeles
US: United States
