# Supplementary material for: Facilitating HIV/AIDS and HIV testing literacy for emergency department patients: a randomized, controlled, trial
Source: BMC Emerg Med. 2018 Jul 9;18:21. doi: 10.1186/s12873-018-0172-7 (PMC6038177; doi:10.1186/s12873-018-0172-7)
Supplement: Supplementary file 1 — Study instruments and video/pictorial brochure elements and script. (DOC 393 kb) [file 12873_2018_172_MOESM1_ESM.doc]

**Supplemental materials**

Included in the supplemental materials are English language copies of the questionnaires, materials, and instruments that will be used for the study, and the HIV/AIDS and HIV testing video and brochure script.

1. Screening, eligibility and enrollment questionnaire
   1. Karliner, et al. English/Spanish language proficiency and preference
   2. Demographic characteristics and nativity
   3. Short Acculturation Scale for Hispanics (SASH) (Hispanics/Latinos only)
   4. HIV testing history and HIV-related exclusion screen
   5. Willingness to undergo rapid HIV testing
2. 3 single-question health literacy screening tests
3. Short Assessment of Health Literacy-English (SAHL-E)
4. HIV testing motivation and behavioral skills questionnaire
5. HIV/AIDS and HIV testing information delivery mode preferences and satisfaction questionnaire
6. HIV/AIDS and HIV testing knowledge questionnaire
7. HIV risk-taking questionnaire
8. HIV test utilization questionnaire
9. Repeat rapid HIV testing acceptance questionnaire
10. Video content organized by Information-Motivation-Behavioral Skills (IMB) model components
11. HIV/AIDS and HIV testing video/brochure script (English and Spanish)

Screening, eligibility and enrollment questionnaire

**Karliner, et al. two-question sequence screen**

**for English/Spanish-language proficiency and preference**

1. **How well do you speak English?**

0 Not at all

1 Not well

2 Well

3 Very well

97 Don't Know

98 Refuse to Answer

99 Research Assistant only

**[If Q1< 2 participant is Spanish-speaking; If Q1=2 go to Q2; Otherwise, continue screening]**

1. **In what language do you prefer to receive your medical care?**

0 Spanish only

1 English

2 Both equally English/Spanish

97 Don't Know

98 Refuse to Answer

99 Research Assistant only

**[If Q2=0 participant is Spanish-speaking; If Q2≥1 participant is English-speaking]**

**Demographic characteristics and nativity**

**1. What is your age?**

**2. What is your gender?**

1 Male

2 Female

3 Transgender (identify with male)

4 Transgender (identify with female)

97 Don’t Know

98 Refuse to Answer

**3. Have you ever been married?** (If yes: Are you currently married?—If yes: Are you living with your spouse? If no: Are you divorced, widowed, or separated? If no: Are you living with anyone else as a partner/domestic partner/couple?) (If no: Are you living with anyone as a partner/domestic partner/couple?) (Choose one)

1 Married

2 Domestic Partner

3 Divorced

4 Widowed

5 Separated

6 Never married

7 Unmarried couple

97 Don't Know

98 Refuse to Answer

**4. What is the highest grade or year of school you completed?** (Choose one)

*[If participant has a GED ask how many years of school were completed]*

1 No School/Kindergarten

2 Grades 1-8 (elementary)

3 Grades 9-11 (some high school)

4 Grade 12 or GED

5 College 1-3 years (some college)

6 College 4 years (college grad)/>College

97 Don't Know

98 Refuse to Answer

**5. Do you have Health Insurance? What Kind do you have? And is that through an employer or a spouse's employer? Or is it from the state?** (Choose one)

1 Private

2 Governmental

3 Private and Governmental

4 None

97 Don't Know

98 Refuse to Answer

**6. Do you consider yourself Hispanic/Latino?**

1 Yes

0 No

97 Don't Know

98 Refuse to Answer

**[If Q6=1, then 6.a., otherwise proceed to 7]**

**6.a. Which one of these groups would you say best represents your race? (Choose one)**

1 White Hispanic

2 Black Hispanic

9 Other

97 Don't Know

1. Refuse to Answer

**7. Which one of these groups would you say best represents your race? (Choose one)**

1. White
2. Black
3. Asian
4. Alaskan native
5. American Indian/Native American
6. Pacific Islander
7. Don't Know

98 Refuse to Answer

**7.a. Other race: ____________________**

**8. Were you born in the United States (excluding Puerto Rico)?**

1 Yes

2 No

97 Don't know

98 Refuse to answer

**[If 8=1, then 8.a., IF 8=1, proceed to 9]**

**8.a. Where were you born?**

1 Argentina

2 Bolivia

3 Canada

4 Chile

5 Colombia

6 Costa Rica

7 Cuba

8 Dominican Republic

9 Ecuador

10 El Salvador

11 Guatemala

12 Honduras

13 Mexico

14 Nicaragua

15 Panama

16 Paraguay

17 Peru

18 Puerto Rico

19 Uruguay

20 Venezuela

21 Spain

22 Other

97 Don't know

98 Refuse to answer

**8.b. Other_______________**

**8.c. How many years in total have you lived in the United States? Count all the years you have lived here, even if you went to your country and returned to the United States.**

1 Less than one year

2 One year to less than two years

3 Two years to less than five years

4 Five years to less than ten years

5 Ten years or more

97 Don't know

98 Refuse to answer

**9. Were either of your parents born outside of the United States?**

1 Yes

2 No

97 Don't know

98 Refuse to answer

**The Short Acculturation Scale for Hispanics (SASH)**

**1. In general, what language(s) do you read and speak?**

1 Only Spanish

2 More Spanish than English

3 Both equally

4 More English than Spanish

5 Only English

**2. What was the language(s) you used as a child?**

1 Only Spanish

2 More Spanish than English

3 Both equally

4 More English than Spanish

5 Only English

**3. What language(s) do you usually speak at home?**

1 Only Spanish

2 More Spanish than English

3 Both equally

4 More English than Spanish

5 Only English

**4. In which language(s) do you usually think?**

1 Only Spanish

2 More Spanish than English

3 Both equally

4 More English than Spanish

5 Only English

**5. What language(s) to you usually speak with your friends?**

1 Only Spanish

2 More Spanish than English

3 Both equally

4 More English than Spanish

5 Only English

**6. In what language(s) are the T.V. programs you usually watch?**

1 Only Spanish

2 More Spanish than English

3 Both equally

4 More English than Spanish

5 Only English

**7. In what language(s) are the radio programs you usually listen to?**

1 Only Spanish

2 More Spanish than English

3 Both equally

4 More English than Spanish

5 Only English

**8. In general, what language(s) are the movies, T.V. and radio programs you prefer to watch and listen to?**

1 Only Spanish

2 More Spanish than English

3 Both equally

4 More English than Spanish

5 Only English

**9. Your close friends are**

1 All Latinos/Hispanics

2 More Latinos than Americans

3 About half and half

4 More Americans than Latinos

5 All Americans

**10. You prefer going to social gatherings/parties at which people are**

1 All Latinos/Hispanics

2 More Latinos than Americans

3 About half and half

4 More Americans than Latinos

5 All Americans

**11. The persons you visit or who visit you are**

1 All Latinos/Hispanics

2 More Latinos than Americans

3 About half and half

4 More Americans than Latinos

5 All Americans

**12. If you could choose your children’s friends you would want them to be**

1 All Latinos/Hispanics

2 More Latinos than Americans

3 About half and half

4 More Americans than Latinos

5 All Americans

**HIV testing history and HIV-related exclusion screen**

1. **Have you ever donated blood?**

1 Yes

0 No

97 Don’t Know

98 Refuse to answer

**[If 1=YES]**

1. **When was the last time you donated blood?**

1 More than 5 years ago

2 Less than 5 years ago, but more than 2 years ago

3 Less than 2 years ago, but more than 1 year ago

4 Less than 1 year, but more than 6 months ago

5 Less than 6 months ago

97 Don't Know

98 Refuse to Answer

**[If 1=YES]**

1. **Except for tests you may have had as part of blood donations, have you ever been tested for HIV?**

1 Yes

0 No

97 Don't Know

98 Refuse to Answer

**[If 1=NO]**

1. **Have you ever been tested for HIV?**

1 Yes

0 No

97 Don’t Know

98 Refuse to Answer

**[If 3=YES]**

1. **Not including blood donations, what was the MAIN reason for your last HIV test?**

1 Exposure from sex

2 Exposure from drugs

3 Exposure from work

4 Medical exam, hospitalization or before surgery

5 Illness

6 Pregnancy

7 Health/Life insurance

8 Military requirement

9 Jail/prison

10 Getting married

11 Blood or blood product transfusion

12 For immigration/visa

13 Just wanted to know

14 Some other reason

97 Don't Know

98 Refuse to Answer

**[If 4=YES]**

1. **What was the MAIN reason for your last HIV test?**

1 Exposure from sex

2 Exposure from drugs

3 Exposure from work

4 Medical exam, hospitalization or before surgery

5 Illness

6 Pregnancy

7 Health/Life insurance

8 Military requirement

9 Jail/prison

10 Getting married

11 Blood or blood product transfusion

12 For immigration/visa

13 Just wanted to know

14 Some other reason

97 Don't Know

98 Refuse to Answer

**[If 3=NO or 4=NO]**

1. **What is the MAIN reason you have NOT been tested for HIV?**

1 Not at risk/not necessary

2 Scared I may have HIV

3 Worried about confidentiality

4 Don't know where/how to get tested

5 No time

6 Don't like needles/having blood taken

7 Hadn't considered it

8 Some other reason

9 Never been asked or offered a test

10 I didn’t have the money for an HIV test

11 I didn’t have transportation to get an HIV test

97 Don't Know

98 Refuse to Answer

**[If 3=YES]**

1. **Not including blood donations, how long has it been since your last HIV test?**

1 More than 5 years ago

2 Less than 5 years ago, but more than 2 years ago

3 Less than 2 years ago, but more than 1 year ago

4 Less than 1 year, but more than 6 months ago

5 Less than 6 months ago

97 Don't Know

98 Refuse to Answer

**[If 4=YES]**

1. **How long has it been since your last HIV test?**

1 More than 5 years ago

2 Less than 5 years ago, but more than 2 years ago

3 Less than 2 years ago, but more than 1 year ago

4 Less than 1 year, but more than 6 months ago

5 Less than 6 months ago

97 Don't Know

98 Refuse to Answer

**[If 1=YES, 3=YES or 4=YES]**

1. **Have you ever tested positive for HIV?**

1 Yes

2 No

97 Don’t Know

98 Refuse to Answer

**[IF 10=1 or 98, then end screening]**

1. **Are you currently participating in an HIV vaccine trial?**

1 Yes

0 No

97 Don't Know

98 Refuse to Answer

**[If Q11=1 or 98 end screening]**

1. **Are you taking medications to keep you from having an HIV infection (PrEP)?**

1 Yes

0 No

97 Don't Know

98 Refuse to Answer

**[If Q12=1 or 98 end screening]**

1. **Are you a part of any study that tests you for HIV? If so, please tell me about it.**

1 Yes

0 No

97 Don't Know

98 Refuse to Answer

**[If Q12=1 or 98 end screening]**

**Willingness to undergo rapid HIV testing**

There are many different types of HIV tests. One type of HIV test is called a rapid HIV test. For a fingerstick rapid HIV test, we will take a drop of blood from your finger. We will then perform the test. The rapid HIV test takes about 20 minutes to perform. You will receive your test results today in the emergency department.

As part of this study, we will ask you to have a rapid HIV test. If your rapid HIV test result is preliminary positive, you will need additional tests to confirm this result. We will help you arrange for these additional tests, as well as any help you might need in getting your final test result. If your rapid HIV test result is preliminary positive, you will not be able to be a part of this study.

If your rapid HIV test is negative, we would invite you to be a part of a one-year study. We will tell you more about that study in a few minutes.

In a few minutes, we will tell you more about the study and give you more information about HIV/AIDS and HIV testing. Right now, we would just like to find out if you are willing to be tested for HIV to see if you can be a part of the study.

1. **Would you like to have a rapid HIV test as part of this study?**

1 Yes

0 No

97 Don’t know

98 Refuse to answer

**[IF QUESTION 1=1]**

**1a. What is the MAIN REASON why you would like to have a rapid HIV test today? (Choose one)**

1 Concerned about having HIV because of a sex or drug exposure

2 Convenient to be tested now

3 I want to be a part of the study

4 Nothing better to do/bored

5 The test is free

6 Because you asked

97 Don’t know

98 Refuse to answer

**[IF QUESTION 1=2]**

**1b. Please tell us the MAIN REASON why you do not want to have an rapid HIV test today?**

**(Choose one)**

1 I do not believe that I am at risk for HIV

2 I do not feel well enough to be tested

3 I think being tested for HIV is too stressful

4 I was recently tested for HIV

5 Family/others are present

6 Worried about confidentiality

7 I don’t want to know if I have HIV

8 I plan to be tested in the future

97 Don’t know

98 Refuse to answer

**[If 1=2 or 97 or 98, then end participation in study]**

3 single-question health literacy screening tests

**3 separate single-question health literacy screening tests**

1. **How often do you have someone, like a family member, friend, hospital or clinic worker, a caregiver or anyone else, help you read materials given to you by the hospital, clinic, or your healthcare provider?**
2. Most of the time
3. A little of the time
4. Some of the time
5. None of the time

97 Don’t know

98 Refuse to answer

1. **How often do you have problems learning about your medical condition or health because of difficulty reading and understanding written information given to you by the hospital, clinic, or your healthcare provider?**
2. Most of the time

2 A little of the time

1 Some of the time

0 None of the time

97 Don’t know

98 Refuse to answer

**3. How confident are you in filling out medical forms by yourself?**

0 Not at all

1 A little bit

2 Somewhat

3 Quite a bit

4 Extremely

97 Don’t know

98 Refuse to answer

Short Assessment of Health Literacy-English (SAHL-E)

**Short Assessment of Health Care Literacy-English (SAHL-E)**

**Interviewer’s Instruction**

The *Short Assessment of Health Literacy-English*, or *SAHL-E*, contains 18 test items designed to assess an English-speaking adult’s ability to read and understand common medical terms. The test could help health professionals estimate the adult’s health literacy level. Administration of the test could facilitated by using laminated 4”×5” flash cards, with each card containing a medical term printed in boldface on the top and the two association words—i.e., the key and the distracter—at the bottom.

**Directions to the Interviewer:**

1. Before the test, the interviewer should say to the examinee:

***“I’m going to show you cards with 3 words on them. First, I’d like you to read the top word out loud. Next, I’ll read the two words underneath and I’d like you to tell me which of the two words is more similar to or has a closer association with the top word. If you don’t know, please say ‘I don’t know’. Don’t guess.”***

1. Show the examinee the first card.
2. The interviewer should say to the examinee:

***“Now, please, read the top word out loud.”***

1. The interviewer should have a clipboard with a score sheet to record the examinee’s answers. The clipboard should be held such that the examinee cannot see or be distracted by the scoring procedure.
2. The interviewer will then read the key and distracter (the two words at the bottom of the card) and then say:

***“Which of the two words is most similar to the top word? If you don’t know the answer, please say ‘I don’t know’.”***

1. The interviewer may repeat the instructions so that the examinee feels comfortable with the procedure.
2. Continue the test with the rest of the cards.
3. A correct answer for each test item is determined by both correct pronunciation and accurate association. Each correct answer gets one point. Once the test is completed, the interviewer should tally the total points to generate the *SAHL-E* score.
4. A score between 0 and 14 suggests the examinee has low health literacy.

The 18 items of *SAHL-E*, rank-ordered according to the parameter *b* of item difficulty (keys and distracters are listed in the same random order as in the field interview)

| Stem | **Key or Distracter** | |  |
| --- | --- | --- | --- |
| 1. kidney | __urine | __fever | __don’t know |
| 1. occupation | __work | __education | __don’t know |
| 1. medication | __instrument | __treatment | __don’t know |
| 1. nutrition | __healthy | __soda | __don’t know |
| 1. miscarriage | __loss | __marriage | __don’t know |
| 1. infection | __plant | __virus | __don’t know |
| 1. alcoholism | __addiction | __recreation | __don’t know |
| 1. pregnancy | __birth | __childhood | __don’t know |
| 1. seizure | __dizzy | __calm | __don’t know |
| 1. dose | __sleep | __amount | __don’t know |
| 1. hormones | __growth | __harmony | __don’t know |
| 1. abnormal | __different | __similar | __don’t know |
| 1. directed | __instruction | __decision | __don’t know |
| 1. nerves | __bored | __anxiety | __don’t know |
| 1. constipation | __blocked | __loose | __don’t know |
| 1. diagnosis | __evaluation | __recovery | __don’t know |
| 1. hemorrhoids | __veins | __heart | __don’t know |
| 1. syphilis | __contraception | __condom | __don’t know |

HIV testing motivation and behavioral skills questionnaire

**HIV testing motivation and behavioral skills questionnaire**

We now would like to ask your opinion about testing for HIV in the emergency department. We also would like to ask you what you think how likely it is that you are risk for currently being infected with HIV. Afterwards, we would like to ask you some questions about HIV testing and how confident you feel about knowing when and how to be tested for HIV.

**Motivation**

1. **How important is it for people to be tested for HIS?**

1 Not at all important

2 A little important

3 Somewhat important

4 Important

5 Very important

97 Don’t know

98 Refuse to answer

1. **How important is it for YOU to be tested for HIV?**

1 Not at all important

2 A little important

3 Somewhat important

4 Important

5 Very important

97 Don’t know

98 Refuse to answer

1. **How much do you think you are at risk for currently being infected with HIV?**
2. I am very much at risk
3. I am pretty much at risk
4. I am somewhat at risk
5. I am not much at risk
6. I am not at risk

97 Don't Know

98 Refuse to Answer

**Behavioral skills**

1. **Think about the following situation. Let’s say that you had sex without a condom last week with someone who has HIV. Yesterday, you were tested for HIV and your antibody test was negative. So, when should you get tested again for HIV?**
2. Since my test was negative, I do not need to be tested again unless I have sex with that person again
3. In one month
4. In two months or sooner if you have sex with that person again.
5. In about three to six months
6. In one year
7. Don’t know
8. Refuse to answer
9. **Think about the following situation. You have been in a relationship with someone for about three months. He/she tells you that he/she got tested for HIV last month and was negative. You and he/she have been having sex without condoms.**
10. You do not need to be tested for HIV since his/her test was negative.
11. Since this is a new relationship, you can wait about one year to be tested for HIV.
12. If you get tested for HIV, you are showing that you do not trust him/her, so you should wait until you find out if this relationship will last.
13. You can wait until your doctor recommends that you be tested for HIV.
14. You should be tested for HIV since you have been having sex with him/her without using condoms.
15. Don’t know
16. Refuse to answer
17. **Think about the following situation. You got tested today for HIV and your rapid HIV antibody test is negative. What does that mean?**
18. This means HIV was found in your system.
19. This means this test result is false positive.
20. This means this test did not find HIV in your system.
21. This means you are infected with HIV.
22. Don’t know
23. Refuse to answer
24. **How confident do you feel that you know when you should get an HIV test?**
25. Not confident at all
26. Somewhat confident
27. Mostly confident
28. Confident
29. Very confident
30. Don’t know
31. Refuse to answer
32. **How confident do you feel about what you need to do to get an HIV test?**
33. Not confident at all
34. Somewhat confident
35. Mostly confident
36. Confident
37. Very confident
38. Don’t know
39. Refuse to answer
40. **How confident do you feel about knowing what your HIV test results mean?**
41. Not confident at all
42. Somewhat confident
43. Mostly confident
44. Confident
45. Very confident
46. Don’t know
47. Refuse to answer

HIV/AIDS and HIV testing information delivery mode preferences and satisfaction questionnaire

**HIV/AIDS and HIV testing information delivery mode**

**preference and satisfaction questionnaire**

**Pre- and post-information delivery mode preferences**

1. **If you had a choice about HOW you got information about HIV/AIDS and HIV testing, would you prefer getting information from:**

1 A brochure with pictures

2 A video

3 Either a video or a brochure with pictures

97 Don’t know

1. Refuse to answer

**Post-information satisfaction**

1. **After you received information about rapid HIV testing from (the video/brochure with pictures), how well informed did you feel?**

1 Not well informed

2 Somewhat informed

3 Well informed

4 Very well informed

97 Don’t know

98 Refuse to answer

HIV/AIDS and HIV testing knowledge questionnaire

**HIV/AIDS and HIV testing knowledge questionnaire**

HIV/AIDS BASICS

1. **If you become infected with HIV, can you completely remove the virus from your body by**

**taking**

medications?

1. No
2. Yes

97 Don’t know

98 Refuse to answer

1. **Do you have to be infected with HIV to get AIDS?**
2. No
3. Yes

97 Don’t know

98 Refuse to answer

1. **Can you always tell if someone has HIV by the way they look?**
2. No
3. Yes

97 Don’t know

98 Refuse to answer

1. **Can a woman who is no longer having her period become infected with HIV?**
2. No
3. Yes

97 Don’t know

98 Refuse to answer

1. **Is being infected with HIV different than having AIDS?**

0 No

1 Yes

97 Don’t know

98 Refuse to answer

TRANSMISSION

1. **Can you get infected with HIV from someone who is infected with HIV but has no**

**symptoms?**

0 No

1 Yes

97 Don’t know

98 Refuse to answer

1. **If someone with HIV kisses you, can you become infected with HIV?**

0 No

1 Yes

97 Don’t know

98 Refuse to answer

1. **If a mother has HIV, is her breast milk safe for her child?**

0 No

1 Yes

97 Don’t know

98 Refuse to answer

1. **Is it possible to be infected with HIV for many years and not know it?**

0 No

1 Yes

97 Don’t know

98 Refuse to answer

1. **If a mosquito bites you after biting someone that has HIV, can you get infected with HIV?**

0 No

1 Yes

97 Don’t know

98 Refuse to answer

PREVENTION

1. **Can you prevent an HIV infection by using a mask over your mouth and nose?**

0 No

1 Yes

97 Don’t know

98 Refuse to answer

1. **Can you prevent an HIV infection using only new needles to inject drugs?**

0 No

1 Yes

97 Don’t know

98 Refuse to answer

1. **Can washing your genitals and private parts help prevent an HIV infection?**

0 No

1 Yes

97 Don’t know

98 Refuse to answer

1. **Can you become infected with HIV by using the same bathroom as someone who has**

**HIV?**

0 No

1 Yes

97 Don’t know

98 Refuse to answer

1. **If your partner has HIV, can you prevent getting infected by using condoms?**

0 No

1 Yes

97 Don’t know

98 Refuse to answer

TYPES OF TESTS

1. **Is it necessary to wait 1 to 2 week to receive the results of a rapid HIV test?**

0 No

1 Yes

97 Don’t know

98 Refuse to answer

1. **If someone has HIV and takes medications to treat it, will this make him/her live a**

**shorter life?**

0 No

1 Yes

97 Don’t know

98 Refuse to answer

1. **Can oral fluids be used for an HIV test?**

0 No

1 Yes

97 Don’t know

98 Refuse to answer

1. **If the person you are having sex with tells you that he/she does not have HIV, should you get tested anyway?**

0 No

1 Yes

97 Don’t know

98 Refuse to answer

1. **If a pregnant woman has HIV, will her baby definitely become infected with HIV, too?**

0 No

1 Yes

97 Don’t know

98 Refuse to answer

TEST RESULTS

1. **If you became infected with HIV one week ago, can your HIV test result be negative?**

0 No

1 Yes

97 Don’t know

98 Refuse to answer

1. **If your HIV test result is negative, does this mean that it is impossible for you to become infected with HIV in the future?**

0 No

1 Yes

97 Don’t know

98 Refuse to answer

1. **Does a preliminary positive test result mean that you can possibly infect others with**

**HIV?**

0 No

1 Yes

97 Don’t know

98 Refuse to answer

1. **Is a special HIV test only necessary for those who have been infected with HIV for many**

**years?**

0 No

1 Yes

97 Don’t know

98 Refuse to answer

1. **If your final HIV test result is positive, can this test result change to negative if you are test for HIV again in 3 months?**

0 No

1 Yes

97 Don’t know

98 Refuse to answer

HIV risk-taking questionnaire

HIV risk-taking questionnaire

**Injection-drug Behaviors**

***We will now ask you if you injected with any drug for non-medical use in the past 3 months.***

***By “non-medical use” we mean using injecting drugs to get high or to help take away the bad effects of other drugs. Injection drugs may be “street drugs” such as heroin, cocaine, crystal meth, or anabolic steroids. Or, injection drugs could be drugs that were prescribed, but were not meant to be injected, such as Percocet.***

**I1.** In the **past 3 months**, have you injected any drugs for non-medical use?

1 Yes

0 No

97 Don’t Know

98 Refuse to answer

**[IF YES TO INJECTION-DRUG USE IN PAST 3 MONTHS (I1)]**

***We will now ask you about sharing needles and syringes and using unclean needles or syringes. By sharing needles or syringes we mean using a needle or syringe after someone else has used it. By unclean needles or syringes we mean needles or syringes that had not been cleaned with bleach and water before they were used.***

**12.** When you injected drugs in the **past 3 months**, how often did you **share** needles or syringes?

3 All of the time

2 Most of the time

1 Some of the time

0 None of the time

97 Don’t Know

98 Refuse to answer

**[If 12 >0]**

**I3**. When sharing needles or syringes in the **past 3 months**, how often did you share **unclean** needles or syringes?

3 All of the time

2 Most of the time

1 Some of the time

0 None of the time

97 Don’t Know

98 Refuse to answer

**1F: Female-only Sexual Behaviors**

***We will now ask you if you have had any male partners in the past 3 months.***

***We will ask you if you had main partners, casual partners, or exchange partners.***

- ***By main partners, we mean men you felt committed to such as boyfriends, husbands, significant others or life partners***
- ***By casual partners, we mean men you had sex with, but did not feel committed to***
- ***By exchange partners, we mean men you gave money, drugs, or other things to pay for sex, or men you had sex with so they would give you money, drugs, or other things***

***For these questions, vaginal sex is when a man puts his penis inside your vagina. Anal sex is when a man puts his penis inside your butt.***

**1F0.** In the **past 3 months**, have you had any male partners?

1 Yes

0 No

97 Don’t Know

98 Refuse to answer

**[IF YES to ANY MALE PARTNERS IN PAST 3 MONTHS (1F0)]**

**1F1.** In the **past 3 months**, have you had any **main male partners**?

**By main male partners we mean men you felt committed to such as boyfriends, husbands, significant others or life partners.**

1 Yes

0 No

97 Don’t Know

98 Refuse to answer

**1F2.** In the **past 3 months**, have you had any **casual male partners**?

**By causal partners, we mean men you had sex with, but did not feel committed to.**

1 Yes

0 No

97 Don’t Know

98 Refuse to answer

**1F3.** In the **past 3 months**, have you had any **exchange male partners**?

**By exchange partners we mean men you gave money, drugs or other things to pay for sex, or men you had sex with so they would give you money, drugs or other things.**

1 Yes

0 No

97 Don’t Know

98 Refuse to answer

**[IF YES to MAIN PARTNERS IN PAST 3 MONTHS (1F1)]**

**2F-MAIN PARTNERS**

**We will now ask you about the main male partners you had in the past 3 months. Please remember, by main partners we mean men you felt committed to, such as boyfriends, husbands, significant others, or life partners.**

**2F1.** In the **past 3 months**, how many **main male partners** did you have?

[1-999]=Range

**[If number of MAIN PARTNERS IN PAST 3 MONTHS=1 (2F1)]**

**2F2.** In the **past 3 months** did you have vaginal and/or anal sex with your **main male partner**?

1 Yes

0 No

97 Don’t Know

98 Refuse to answer

**[If YES to SEX WITH MAIN MALE PARTNER IN PAST 3 MONTHS=1 (2F2)]**

**2F3.** When you had vaginal and/or anal sex in the **past 3 months** with your **main male partner**, how often did he use condoms?

3 None of the time

2 Some of the time

1 Most of the time

0 All of the time

97 Don’t Know

98 Refuse to answer

**[If # MAIN PARTNERS IN PAST 3 MONTHS>1 (2F1)]**

**2F2a.** In the **past 3 months** did you have vaginal and/or anal sex with **any** of your **main male partners**?

1 Yes

0 No

97 Don’t Know

98 Refuse to answer

**[If YES to SEX WITH MAIN MALE PARTNERS IN PAST 0 MONTHS (2F2a)]**

**2F3a.** When you had vaginal and/or anal sex in the **past 3 months** with your **main male partners**, how often did **they** use condoms?

3 None of the time

2 Some of the time

1 Most of the time

0 All of the time

97 Don’t Know

98 Refuse to answer

**[IF YES to CASUAL PARTNERS IN PAST 3 MONTHS (1F2)]**

**3F-CASUAL PARTNERS**

**We will now ask you about the casual male partners you had in the past 3 months. Please remember, by casual partners we mean men you had sex with, but did not feel committed to.**

**3F1.** In the **past 3 months**, how many **casual male partners** did you have?

[1-999]=Range

**[If # CASUAL PARTNERS IN PAST 3 MONTHS=1 (3F1)]**

**3F2.** In the **past 3 months** did you have vaginal and/or anal sex with your **casual male partner**?

1 Yes

0 No

97 Don’t Know

98 Refuse to answer

**[If YES to SEX WITH CASUAL MALE PARTNER IN PAST 3 MONTHS (3F2)]**

**3F3.** When you had vaginal and/or anal sex in the **past 3 months** with your **casual male partner**, how often did he use condoms?

3 None of the time

2 Some of the time

1 Most of the time

0 All of the time

97 Don’t Know

98 Refuse to answer

**[If # CASUAL PARTNERS IN PAST 3 MONTHS>1 (3F1)]**

**3F2a.** In the **past 3 months** did you have vaginal and/or anal sex with any of your **casual male partners**?

1 Yes

0 No

97 Don’t Know

98 Refuse to answer

**[If YES to SEX WITH CASUAL MALE PARTNERS IN PAST 3 MONTHS (3F2a)]**

**3F3a.** When you had vaginal and/or anal sex in the **past 3 months** with your **casual male partners**, how often did **they** use condoms?

3 None of the time

2 Some of the time

1 Most of the time

0 All of the time

97 Don’t Know

98 Refuse to answer

**[IF YES to EXCHANGE PARTNERS IN PAST 3 MONTHS (1F3)]**

**4F-EXCHANGE PARTNERS**

**We will now ask you about the exchange male partners you had vaginal and/or anal sex with in the past 3 months. Please remember, by exchange partners we mean men you gave money, drugs or other things to pay for sex or men you had sex with so they would give you money, drugs or other things.**

**4F1.** In the **past 3 months**, how many **exchange male partners** did you have?

[1-999]=Range

**[If # EXCHANGE PARTNERS IN PAST 4 MONTHS=1 (4F1)]**

**4F2.** In the **past 3 months** have you had vaginal and/or anal sex with your **exchange male partner**?

1 Yes

0 No

97 Don’t Know

98 Refuse to answer

**[If YES to SEX WITH EXCHANGE MALE PARTNER IN PAST 3 MONTHS (4F2)]**

**4F3.** When you had vaginal and/or anal sex in the **past 3 months** with your **exchange male partner**, how often did he use condoms?

3 None of the time

2 Some of the time

1 Most of the time

0 All of the time

97 Don’t Know

98 Refuse to answer

**[If # EXCHANGE PARTNERS IN PAST 3 MONTHS>1 (4F1)]**

**4F2a.** In the **past 3 months** did you have vaginal and/or anal sex with any of your **exchange male partners**?

1 Yes

0 No

97 Don’t Know

98 Refuse to answer

**[If YES to SEX WITH EXCHANGE MALE PARTNERS IN PAST 3 MONTHS (4F2a)]**

**4F3a.** When you had vaginal and/or anal sex in the **past 3 months** with your **exchange male partners**, how often did **they** use condoms?

3 None of the time

2 Some of the time

1 Most of the time

0 All of the time

97 Don’t Know

98 Refuse to answer

**1MF: Male-Female Sexual Behaviors**

***We will now ask you if you have had any female partners in the past 3 months.***

***We will ask you if you had main partners, casual partners, or exchange partners.***

- ***By main partners, we mean women you felt committed to such as girlfriends, wives, significant others, or life partners.***
- ***By casual partners, we mean women you had sex with, but did not feel committed to.***
- ***By exchange partners, we mean women you gave money, drugs, or other things to pay for sex, or women you had sex with so they would give you money, drugs, or other things.***

For these questions, vaginal sex is when YOU put YOUR penis inside a woman’s vagina. Anal sex is when YOU put YOUR penis inside of a woman's butt.

**1MF0.** In the **past 3 months**, did you had any **female partners**?

1 Yes

0 No

97 Don’t Know

98 Refuse to answer

**[IF YES to ANY FEMALE PARTNERS IN PAST 3 MONTHS (1MF0)]**

**1MF1.** In the **past 3 months**, did you have any **main female partners**?

***By main female partners we mean women you felt committed to such as girlfriends, wives, significant others or life partners.***

1 Yes

0 No

97 Don’t Know

98 Refuse to answer

**1MF2.** In the **past 3 months**, did you have any **casual female partners**?

***By causal partners, we mean women you had sex with, but did not feel committed to.***

1 Yes

0 No

97 Don’t Know

98 Refuse to answer

**1MF3.** In the **past 3 months**, did you have any **exchange female partners**?

***By exchange partners we mean women you gave money, drugs, or other things to pay for sex, or women you had sex with so they would give you money, drugs, or other things.***

1 Yes

0 No

97 Don’t Know

98 Refuse to answer

**[IF YES to MAIN PARTNERS IN PAST 3 MONTHS (1MF1)]**

**2MF-MAIN PARTNERS**

**We will now ask you about the main female partners you had in the past 3 months. Please remember, by main partners we mean women you felt committed to such as girlfriends, wives, significant others or life partners.**

**2MF1.** In the **past 3 months**, how many **main female partners** did you have?

[1-999]=Range

**[IF MAIN PARTNER IN PAST 3 MONTHS=1 (2MF1)]**

**2MF2.** In the **past 3 months** did you have vaginal and/or anal sex with your **main female partner**?

1 Yes

0 No

97 Don’t Know

98 Refuse to answer

**[If YES to SEX WITH MAIN FEMALE PARTNER IN PAST 3 MONTHS (2MF2)]**

**2MF3.** When you had vaginal and/or anal sex in the **past 3 months** with your **main female partner**, how often did **you** use condoms?

3 None of the time

2 Some of the time

1 Most of the time

0 All of the time

97 Don’t Know

98 Refuse to answer

**[If # MAIN PARTNERS IN PAST 3 MONTHS>1 (2MF1)]**

**2MF2a.** In the **past 3 months** did you have vaginal and/or anal sex with any of your **main female partners**?

1 Yes

0 No

97 Don’t Know

98 Refuse to answer

**[If YES to SEX WITH MAIN FEMALE PARTNERS IN PAST 3 MONTHS (2MF2a)]**

**2MF3a.** When you had vaginal and/or anal sex in the **past 3 months** with your **main female partners**, how often did **you** use condoms?

3 None of the time

2 Some of the time

1 Most of the time

0 All of the time

97 Don’t Know

98 Refuse to answer

**[IF YES to CASUAL PARTNERS IN PAST 3 MONTHS (1MF2)]**

**3MF-CASUAL PARTNERS**

**We will now ask you about the casual female partners you had in the past 3 months. Please remember, by CASUAL PARTNERS we mean we mean women you had sex with, but did not feel committed to.**

**3MF1.** In the **past 3 months**, how many **casual female partners** did you have?

Please enter the number and hit “Next Question” to continue.

[1-999]=Range

**[If # CASUAL PARTNERS IN PAST 3 MONTHS=1 (3MF1)]**

**3MF2.** In the **past 3 months** did you have vaginal and/or anal sex with your **casual female partner**?

1 Yes

0 No

97 Don’t Know

98 Refuse to answer

**[If YES to SEX WITH CASUAL FEMALE PARTNER IN PAST 3 MONTHS (3MF2)]**

**3MF3.** When you had vaginal and/or anal sex in the **past 3 months** with your **casual female partner**, how often did **you** use condoms?

3 None of the time

2 Some of the time

1 Most of the time

0 All of the time

97 Don’t Know

98 Refuse to answer

**[If # CASUAL PARTNERS IN PAST 3 MONTHS>1 (3MF1)]**

**3MF2a.** In the **past 3 months** did you have vaginal and/or anal sex with any of your **casual female partners**?

1 Yes

0 No

97 Don’t Know

98 Refuse to answer

**[If YES to SEX WITH CASUAL FEMALE PARTNERS IN PAST 3 MONTHS (3MF2a)]**

**3MF3a.** When you had vaginal and/or anal sex in the **past 3 months** with your **casual female partners**, how often did **you** use condoms?

3 None of the time

2 Some of the time

1 Most of the time

0 All of the time

97 Don’t Know

98 Refuse to answer

**[IF YES to EXCHANGE PARTNERS IN PAST 3 MONTHS (1MF3)]**

**4MF-EXCHANGE PARTNERS**

**We will now ask you about the EXCHANGE FEMALE PARTNERS you had in the past 3 months. Please remember, by EXCHANGE PARTNER, we mean women you gave money, drugs, or other things to pay for sex, or women you had sex with so they would give you money, drugs, or other things**

**4MF1.** In the **past 3 months**, how many **exchange female partners** did you have?

[1-999]=Range

**[If # EXCHANGE PARTNERS IN PAST 3 MONTHS=1 (4MF1)]**

**4MF2.** In the **past 3 months** did you have vaginal and/or anal sex with your **exchange female partner**?

1 Yes

0 No

97 Don’t Know

98 Refuse to answer

**[If YES to SEX WITH EXCHANGE FEMALE PARTNER IN PAST 3 MONTHS (4MF2)]**

**4MF3.** When you had vaginal and/or anal sex in the **past 3 months** with your **exchange female partner**, how often did **you** use condoms?

3 None of the time

2 Some of the time

1 Most of the time

0 All of the time

97 Don’t Know

98 Refuse to answer

**[If # EXCHANGE PARTNERS IN PAST 3 MONTHS>1 (4M4)]**

**4MF2a.** In the **past 3 months** did you have vaginal and/or anal sex with any of your **exchange female partners**?

1 Yes

0 No

97 Don’t Know

98 Refuse to answer

**[If YES to SEX WITH EXCHANGE FEMALE PARTNERS IN PAST 3 MONTHS (4MF2a)]**

**4MF2b.** When you had vaginal and/or anal sex in the **past 3 months** with your **exchange female partners**, how often did **you** use condoms?

3 None of the time

2 Some of the time

1 Most of the time

0 All of the time

97 Don’t Know

98 Refuse to answer

**1MM: Male-Male Sexual Behaviors**

***We will now ask you if you have had any MALE PARTNERS in the past 3 months.***

***We will ask you if you had main partners, casual partners, or exchange partners.***

- ***By main partners, we mean men you felt committed to such as boyfriends, husbands, significant others or life partners***
- ***By casual partners, we mean men you had sex with, but did not feel committed to***
- ***By exchange partners, we mean men you gave money, drugs, or other things to pay for sex, or men you had sex with so they would give you money, drugs, or other things***

For these questions, anal sex is when YOU put YOUR penis inside of a man's butt or when a MAN puts HIS penis inside of YOUR butt. You were the “TOP” if when you had anal sex your penis was inside a man’s butt. You were the “BOTTOM” if when you had anal sex a man put his penis inside your butt.

**1MM0.** In the **past 3 months**, did you have any **male partners**?

1 Yes

0 No

97 Don’t Know

98 Refuse to answer

**[IF YES to ANY MALE PARTNERS IN PAST 3 MONTHS (1MM0)]**

**1MM1.** In the **past 3 months**, did you have any **main male partners**?

**By main male partners we mean men you felt committed to such as boyfriends, husbands, significant others or life partners.**

1 Yes

0 No

97 Don’t Know

98 Refuse to answer

**1MM2.** In the **past 3 months**, did you have any **casual male partners**?

**By causal partners, we mean men you had sex with, but did not feel committed to.**

1 Yes

0 No

97 Don’t Know

98 Refuse to answer

**1MM3.** In the **past 3 months**, did you have any **exchange male partners**?

**By exchange partners we mean men you gave money, drugs or other things to pay for sex, or men you had sex with so they would give you money, drugs or other things.**

1 Yes

0 No

97 Don’t Know

98 Refuse to answer

**[IF YES to MAIN PARTNERS IN PAST 3 MONTHS (1MM1)]**

**2MM-MAIN PARTNERS**

**We will now ask you about the main male partners you had in the past 3 months. Please remember, by main partners we mean men you felt committed to, such as boyfriends, husbands, significant others, or life partners.**

**2MM1.** In the **past 3 months**, how many **main male partners** did you have?

Please enter the number and hit “Next Question” to continue.

[1-999]=Range

**[IF YES to MAIN PARTNER IN PAST 3 MONTHS (2MM1)]**

**2MM2T.** In the **past 3 months** did you have anal sex with your **main male partner when you were the TOP**?

You were the “TOP” if you had anal sex when your penis was inside a man’s butt.

1 Yes

0 No

97 Don’t Know

98 Refuse to answer

**[IF YES to SEX WITH MAIN PARTNER WHEN THE TOP IN PAST 3 MONTHS (2MM2T)]**

**2MM3T.** When you had anal sex in the **past 3 months** with your **main male partner** and you were the **TOP**, how often did **you** use condoms?

4 None of the time

3 Some of the time

2 Most of the time

1 All of the time

97 Don’t Know

98 Refuse to answer

**2MM2B.** In the **past 3 months** did you have anal sex with your **main male partner** when you were the **BOTTOM**? You were the “BOTTOM” if you had anal sex when a man put his penis inside your butt.

1 Yes

0 No

97 Don’t Know

98 Refuse to answer

**[If YES to SEX WITH MAIN MALE PARTNER WHEN THE BOTTOM IN PAST 3 MONTHS (2MM2B)]**

**2MM3B.** When you had anal sex in the **past 3 months** with your **main male partner** and you were the **BOTTOM**, how often did **he** use condoms?

4 None of the time

3 Some of the time

2 Most of the time

1 All of the time

97 Don’t Know

98 Refuse to answer

**[If # MAIN PARTNERS IN PAST 3 MONTHS>1 (2MM1)]**

**2MM2Ta.** In the **past 3 months** did you have anal sex with any of your **main male partners** when you were the **TOP**? You were the “TOP” if you had anal sex when your penis was inside a man’s butt.

1 Yes

0 No

97 Don’t Know

98 Refuse to answer

**[If YES to SEX WITH MAIN MALE PARTNERS IN PAST 3 MONTHS (2MM2Ta)]**

**2MM2Tb.** When you had anal sex in the **past 3 months** with your **main male partners** and you were the **TOP**, how often did **you** use condoms?

4 None of the time

3 Some of the time

2 Most of the time

1 All of the time

97 Don’t Know

98 Refuse to answer

**2MM2Ba.** In the **past 3 months** did you have anal sex with your **main male partners** when you were the **BOTTOM**? You were the “BOTTOM” if you had anal sex when a man put his penis inside your butt.

1 Yes

0 No

97 Don’t Know

98 Refuse to answer

**[If YES to SEX WITH MAIN MALE PARTNERS WHEN THE BOTTOM IN PAST 3 MONTHS (2MM2Ba)]**

**2MM2Bb.** When you had anal sex in the **past 3 months** with your **main male partners** and you were the **BOTTOM**, how often did **they** use condoms?

4 None of the time

3 Some of the time

2 Most of the time

1 All of the time

97 Don’t Know

98 Refuse to answer

**[IF YES to CASUAL PARTNERS IN PAST 3 MONTHS (1MM2)]**

**3MM-CASUAL PARTNERS**

**We will now ask you about the CASUAL MALE PARTNERS you had in the past 3 months. Please remember, by CASUAL MALE PARTNERS we mean men you had sex with, but did not feel committed to.**

**3MM1.** In the **past 3 months**, how many **casual male partners** did you have?

[1-999]=Range

**[IF YES to CASUAL PARTNER IN PAST 3 MONTHS (3MM1)]**

**3MM2T.** In the **past 3 months** did you have anal sex with your **casual male partner when you were the TOP**? You were the “TOP” if you had anal sex when your penis was inside a man’s butt.

1 Yes

0 No

97 Don’t Know

98 Refuse to answer

**[IF YES to SEX WITH CASUAL PARTNER WHEN THE TOP IN PAST 3 MONTHS (3MM2T)]**

**3MM3T.** When you had anal sex in the **past 3 months** with your **casual male partner** and you were the **TOP**, how often did **you** use condoms?

4 None of the time

3 Some of the time

2 Most of the time

1 All of the time

97 Don’t Know

98 Refuse to answer

**3MM2B.** In the **past 3 months** did you have anal sex with your **casual male partner** when you were the **BOTTOM**? You were the “BOTTOM” if you had anal sex when a man put his penis inside your butt.

1 Yes

0 No

97 Don’t Know

98 Refuse to answer

**[If YES to SEX WITH CASUAL MALE PARTNER WHEN THE BOTTOM IN PAST 3 MONTHS (3MM2B)]**

**3MM3B.** When you had anal sex in the **past 3 months** with your **casual male partner** and you were the **BOTTOM**, how often did **he** use condoms?

4 None of the time

3 Some of the time

2 Most of the time

1 All of the time

97 Don’t Know

98 Refuse to answer

**[If # CASUAL PARTNERS IN PAST 3 MONTHS>1 (3MM1)]**

**3MM2Ta.** In the **past 3 months** did you have anal sex with any of your **casual male partners** when you were the **TOP**? You were the “TOP” if you had anal sex when your penis was inside a man’s butt.

1 Yes

0 No

97 Don’t Know

98 Refuse to answer

**[If YES to SEX WITH CASUAL MALE PARTNERS IN PAST 3 MONTHS (3MM2Ta)]**

**3MM2Tb.** When you had anal sex in the **past 3 months** with your **casual male partners** and you were the **TOP**, how often did **you** use condoms?

4 None of the time

3 Some of the time

2 Most of the time

1 All of the time

97 Don’t Know

98 Refuse to answer

**3MM2Ba.** In the **past 3 months** did you have anal sex with your **casual male partners** when you were the **BOTTOM**? You were the “BOTTOM” if you had anal sex when a man put his penis inside your butt.

1 Yes

0 No

97 Don’t Know

98 Refuse to answer

**[If YES to SEX WITH CASUAL MALE PARTNERS WHEN THE BOTTOM IN PAST 3 MONTHS (3MM2Ba)]**

**3MM2Bb.** When you had anal sex in the **past 3 months** with your **casual male partners** and you were the **BOTTOM**, how often did **they** use condoms?

4 None of the time

3 Some of the time

2 Most of the time

1 All of the time

97 Don’t Know

98 Refuse to answer

**[IF YES to EXCHANGE PARTNERS IN PAST 3 MONTHS (1MM3)]**

**4MM-EXCHANGE PARTNERS**

**We will now ask you about the EXCHANGE MALE PARTNERS you had in the past 3 months. Please remember, by EXCHANGE MALE PARTNERS we mean men you had sex with, but did not feel committed to.**

**4MM1.** In the **past 3 months**, how many **exchange male partners** did you have?

[1-999]=Range

**[IF YES to EXCHANGE PARTNER IN PAST 3 MONTHS (3MM1)]**

**4MM2T.** In the **past 3 months** did you have anal sex with your **exchange male partner when you were the TOP**? You were the “TOP” if you had anal sex when your penis was inside a man’s butt.

1 Yes

0 No

97 Don’t Know

98 Refuse to answer

**[IF YES to SEX WITH EXCHANGE PARTNER WHEN THE TOP IN PAST 3 MONTHS (4MM2T)]**

**4MM3T.** When you had anal sex in the **past 3 months** with your **exchange male partner** and you were the **TOP**, how often did **you** use condoms?

4 None of the time

3 Some of the time

2 Most of the time

1 All of the time

97 Don’t Know

98 Refuse to answer

**4MM2B.** In the **past 3 months** did you have anal sex with your **exchange male partner** when you were the **BOTTOM**? You were the “BOTTOM” if you had anal sex when a man put his penis inside your butt.

1 Yes

0 No

97 Don’t Know

98 Refuse to answer

**[If YES to SEX WITH EXCHANGE MALE PARTNER WHEN THE BOTTOM IN PAST 3 MONTHS (4MM2B)]**

**4MM3B.** When you had anal sex in the **past 3 months** with your **exchange male partner** and you were the **BOTTOM**, how often did **he** use condoms?

4 None of the time

3 Some of the time

2 Most of the time

1 All of the time

97 Don’t Know

98 Refuse to answer

**[If # EXCHANGE PARTNERS IN PAST 3 MONTHS>1 (4MM1)]**

**4MM2Ta.** In the **past 3 months** did you have anal sex with any of your **exchange male partners** when you were the **TOP**? You were the “TOP” if you had anal sex when your penis was inside a man’s butt.

1 Yes

0 No

97 Don’t Know

98 Refuse to answer

**[If YES to SEX WITH EXCHANGE MALE PARTNERS IN PAST 3 MONTHS (4MM2Ta)]**

**4MM2Tb.** When you had anal sex in the **past 3 months** with your **exchange male partners** and you were the **TOP**, how often did **you** use condoms?

4 None of the time

3 Some of the time

2 Most of the time

1 All of the time

97 Don’t Know

98 Refuse to answer

**4MM2Ba.** In the **past 3 months** did you have anal sex with your **exchange male partners** when you were the **BOTTOM**? You were the “BOTTOM” if you had anal sex when a man put his penis inside your butt.

1 Yes

0 No

97 Don’t Know

98 Refuse to answer

**[If YES to SEX WITH EXCHANGE MALE PARTNERS WHEN THE BOTTOM IN PAST 3 MONTHS (4MM2Ba)]**

**4MM2Bb.** When you had anal sex in the **past 3 months** with your **exchange male partners** and you were the **BOTTOM**, how often did **they** use condoms?

4 None of the time

3 Some of the time

2 Most of the time

1 All of the time

97 Don’t Know

98 Refuse to answer

HIV test utilization questionnaire

**HIV test utilization questionnaire**

1. **In the past 3 months, did you donate blood?**

1 Yes

0 No

97 Don’t know

98 Refuse to answer

1. **In the past 3 months, other than blood donations, were you tested for HIV?**

1 Yes

0 No

97 Don’t know

98 Refuse to answer

**2.a. In the past 3 months when you were tested for HIV, HOW were you given information**

**about HIV/AIDS and HIV testing?**

1 I did not receive any information

2 From a person

3 From a video

4 From a brochure without pictures

5 From a brochure with pictures

97 Don’t know

1. Refuse to answer

**2.b. What was the MAIN reason you were tested for HIV?**

1 Exposure from sex

2 Exposure from drugs

3 Exposure from work

4 Medical exam, hospitalization or before surgery

5 Illness

6 Pregnancy

7 Health/Life insurance

8 Military requirement

9 Jail/prison

10 Getting married

11 Blood or blood product transfusion

12 For immigration/visa

13 Just wanted to know

14 Part of a research study

15 Some other reason

97 Don't know

1. Refuse to answer
2. **In the past 3 months, did you have a positive HIV test?**

1 Yes

0 No

97 Don’t know

98 Refuse to answer

Repeat rapid HIV testing acceptance questionnaire

**Repeat rapid HIV testing acceptance questionnaire**

**Thank you for being a part of our study! We are very grateful for your help. We would now like to offer you the chance to be tested for HIV one more time as part of this study. We would like to offer you a special kind of rapid HIV test. This rapid HIV test is one you can do yourself at home or at any place you choose. For this “home” rapid HIV self-test, you would use a swab of your mouth and test it for HIV using an easy-to-use kit. If you prefer, you can bring the test kit back to us and we will help you use it. The test kit is free to you. If you decide to get tested, we will tell you how to order the test for free by telephone or through the internet.**

1. **Would you like to be tested for HIV again as part of this study?**
2. No, I do not want to be tested for HIV again as part of the study.
3. Yes, I would like to be tested for HIV again. I will use the test kit myself.
4. Yes, I would like to be tested for HIV again. But, I would like to bring the test kit back to you and ask for your help in using it.
5. Don’t know
6. Refuse to answer

**1.a. What is the main reason you do not want to be tested for HIV again?**

1. I am not at risk, so it is not necessary for me to be tested again.
2. I am scared I may have HIV.
3. I have an HIV test scheduled outside of the study.
4. I am tired of being in the study.
5. I have been tested very recently.
6. I do not trust the HIV test you are offering.
7. I am worried someone might find out about the results.
8. I am worried about losing my job, insurance, housing, family or friends if people found out I tested positive.
9. I am worried my name would be reported to the government if I test positive.
10. I do not have time
11. Some other reason

97 Don't Know

98 Refuse to Answer

**1.b. What is the main reason you want to get tested again for HIV?**

1 I think I should be tested regularly for HIV.

2 I am scared I may have HIV.

3 I don’t know if I have HIV.

4 I have had sex with someone without using a condom.

5 I have injected drugs.

6 Some other reason

97 Don't Know

98 Refuse to Answer

**2.a. What is the main reason you chose to do the rapid HIV test yourself?**

1. I would like to try this “home” rapid HIV self-test.
2. I have heard about this “home” rapid HIV self-test and am curious about it.
3. I feel confident that I can test myself.
4. I do not have transportation to get to the testing site.
5. I prefer the privacy of being tested at home or another place of my choice.
6. It is more convenient for me to be tested at home or another place of my choice.
7. Some other reason
8. Don't Know
9. Refuse to Answer

**2.b. What is the main reason you chose to have us help you do the rapid HIV test?**

1. I do not feel confident that I would know how to test myself.
2. I would like to talk with a counselor when I get the test.
3. I am too scared to test myself.
4. I will be more confident in the test results if they are interpreted by a health professional.
5. I do not want the people I live with to see the test arrive.
6. I’m not sure the at-home test would be accurate.
7. I think that a health care professional should test people for HIV.
8. Some other reason
9. Don't Know
10. Refuse to Answer

Video and brochure content organized by IMB model components

**Video and brochure content organized by IMB model components**

**Behavior = HIV Testing**

| **IMB Model Components** | **Video Content Areas/Messages** |
| --- | --- |
|  | General HIV/AIDS knowledge/prevention |
| I | Understanding HIV and AIDS, and the difference |
| I | Infection is invisible and may be undetected |
| I/M | If you are carrying the virus, could infect others |
| I | Anyone can get HIV |
| M | Effect of HIV on immune system over time (worse untreated) |
| I | Transmission Risk |
| -- | Only takes one exposure to be at risk |
| I/M | May pass HIV to a baby during pregnancy, birth, breast feeding |
| I | HIV transmission can be prevented |
| -- | Sexual risk prevention strategies |
| -- | Inject related risk prevention strategies |
|  | Testing specific content areas and messages |
| I | Get tested! Only way to find out if you have HIV |
| I | May not know if you are infected |
| I/M | Early identification helps you stay healthy longer |
| M | Staying healthy impacts your family and friends |
| M | Knowing status impacts your sexual partners and others |
| I | Partner Notifications services often available |
| I/M | Resources available for HIV + people (medical and other) |
| I/M | Medical treatments available to help you live longer |
| I | Blood test versus oral testing option |
| I | Rapid versus other testing options |
| I | Results are important |
| BS | Get your results |
| I | 3-month window period |
| BS | Risk prevention during window period |
| I/BS | Re-testing, general and for potential acute infection |
| I/BS | Understanding test results |
| I/BS | Confirmatory Testing |

I: Information

M: Motivation

BS: Behavioral skills

| HIV/AIDS and HIV testing  video script  (English and Spanish) |
| --- |

| **Video Script – English/Spanish** | |
| --- | --- |
| **IMAGES** | **NARRATIVE** |
| 1.  Yellow background with plus and minus signs.  Screen reads, ¿*Qué sabes sobre el VIH y sobre las pruebas de VIH?*  Yellow background with plus and minus signs.  Screen reads, *What do you know about HIV and HIV testing?* | ¿Qué sabes sobre el VIH y sobre las pruebas de VIH?  What do you know about HIV and HIV testing? |
| 2.  Cartoon torsos of young man (black hair) and woman (red hair) surrounded by question marks and looking up to *¿Qué es el VIH y qué son las pruebas del VIH?*  Cartoon torsos of young man (black hair) and woman (red hair) surrounded by question marks and looking up to *What is HIV and HIV testing?* | Hoy vas a aprender sobre el VIH y sobre las pruebas de VIH. Hablaremos sobre…(pause)  Today you are going to learn about HIV and HIV testing. We will talk about…(pause) |
| 3.  Cartoon torsos surrounded by question marks look directly in front of them. Screen reads, *¿Cuál es la diferencia entre el VIH y el SIDA?*  Cartoon torsos surrounded by question marks look directly in front of them. Screen reads,  *What is the difference between HIV and AIDS?* | La diferencia entre estar infectado con el VIH y tener SIDA.  The difference between being infected with HIV and having AIDS. |
| 4.  Cartoon torsos surrounded by question marks look at each other. Screen reads, *¿Cómo se infecta alguien con el VIH?*  Cartoon torsos surrounded by question marks look at each other. Screen reads, *How do you get HIV?* | Cómo alguien se puede infectar con el VIH…(pause)  How someone can get infected with HIV…(pause) |
| 5.  Cartoon torsos come closer, look at each other and wink. Screen reads, *¿Cómo se puede prevenir el VIH?*  Cartoon torsos come closer, look at each other and wink. Screen reads, *How can HIV be prevented?* | Y sobre cómo se puede prevenir el VIH.  And about how HIV can be prevented. |
| 6.  Dissolve to split frames of cartoon doctors/professionals checking the young man and woman. Screen reads, *Hacerse la prueba trae beneficios*  Dissolve to split frames of cartoon doctors/professionals checking the young man and woman. Screen reads, *Benefits of testing* | También hablaremos sobre porque deberías hacerte una prueba de VIH.  We will also talk about why you should get tested for HIV. |
| 7.  Cartoon of female doctor holding papers and talking to young woman. Screen reads,  *Hablaremos sobre las pruebas*  Dissolve to male doctor talking to young man.  Screen reads, *Explicaremos los resultados*  Cartoon of female doctor holding papers and talking to young woman. Screen reads,  *Talk about testing*  Dissolve to male doctor talking to young man.  Screen reads, *Explain results* | Más adelante, hablaremos sobre algunos tipos de pruebas de VIH y explicaremos lo que significan los resultados de estas pruebas.(pause)  Later, we will talk about some types of HIV tests and explain the meaning of test results. (pause) |
| 8.  Cartoon of young man and woman surrounded by questions marks, who then look at each other. Screen reads, *¿Cuál es la diferencia entre el VIH y el SIDA?*  Cartoon of young man and woman surrounded by questions marks, who then look at each other. Screen reads, *What is the difference between HIV and AIDS?* | Entonces, ¿Cuál es la diferencia entre el VIH y el SIDA?  So, what is the difference between HIV and AIDS? |
| 9.  1. Cartoon of young man and woman surrounded by questions marks looking in front. Screen reads, *¿Qué es el VIH?*  2. At bottom of screen a sign that reads, ***V****irus de la* ***I****nmunodeficiencia* ***H****umana* pops up and man and woman look down to it  1. Cartoon of young man and woman surrounded by questions marks looking in front. Screen reads, *What is HIV?*  2. At bottom of screen a sign that reads, ***H****uman* ***I****mmunodeficiency* ***V****irus* pops up and man and woman look down to it | Las letras V-I-H significan Virus de la Inmunodeficiencia Humana.  The letters H-I-V stand for Human Immunodeficiency Virus. |
| 10.  Top of the Screen: El VIH es un virus  Then transitions to: Un virus es un tipo de organismo  Cartoon of the HIV virus as a blob that reads “*VIH*” in the center of it. Then this image fades to show the cartoon  of one of the main female character (protagonist) with *VIH* blob cartoon on her shirt  Last transitions to: VIH es Permanente  This scene will have a total of 3 screens  Top of the screen: HIV is a virus  Then transitions to: A virus is a type of organism  Cartoon of the HIV virus as a blob that reads “*VIH*” in the center of it. Then this image fades to show the cartoon  of one of the main female character (protagonist) with *VIH* blob cartoon on her shirt  Last transitions to: HIV is Permanent  This scene will have a total of 3 screens | El VIH es un virus. Un virus es un tipo de organismo. A diferencia de otros virus, una vez que te has infectado con el VIH, éste se queda en tu cuerpo para siempre.(pause)  HIV is a virus. A virus is a type of organism. Unlike other viruses, once you are infected with HIV, it stays in your body forever. (pause) |
| 11.  Title: Infectado por toda su vida  Female cartoon character from above is surrounded by a flashing line representing the immune system. The word “*Sistema Inmunológico*” wraps around the line. HIV cartoon blob attacks immune system line, like a pac-man, leaving gaps in between. Line stops flashing.  Her shirt will say VIH  Title: Infected for life  Female cartoon character from above is surrounded by a flashing line representing the immune system. The word “*Immune System*” wraps around the line. HIV cartoon blob attacks immune system line, like a pac-man, leaving gaps in between. Line stops flashing.  Her shirt will say HIV | Cuando te infectas con el VIH, el virus daña tu sistema inmunológico. Tu sistema inmunológico defiende tu cuerpo contra las enfermedades.  When you are infected with HIV, the virus damages your immune system. Your immune system defends your body against diseases. |
| 12.  Word *VIH* appears on the screen, then an arrow next to it and finally the word *SIDA* appears at the other end of the arrow.  Word *HIV* appears on the screen, then an arrow next to it and finally the word *AIDS* appears at the other end of the arrow. | El VIH es el virus que causa el SIDA. (quick pause)  HIV is the virus that causes AIDS. (quick pause) |
| 13.  Top of screen: ¿Que es el SIDA? This fades away when letters at the bottom of screen appear  At bottom of screen a sign that reads, ***S****índrome de* ***In****munodeficiencia* ***A****dquirida* pops up and man and woman look down to it.  Top of screen: What is AIDS? This fades away when letters at the bottom of screen appear  At bottom of screen a sign that reads, ***A****cquired* ***I****mmune* ***D****eficiency* ***S****yndrom****e*** pops up and man and woman look down to it. | Las letras en la palabra SIDA significan Síndrome de Inmunodeficiencia Adquirida.  The letters in A-I-D-S stand for the Acquired Immune Deficiency Syndrome. |
| 14.  Two blocks linked by a chain, one reading *VIH* and one reading *SIDA* pop up at the top of the screen. Man and woman, still surrounded by question marks, look at each other and then to the front.  Two blocks linked by a chain, one reading *HIV* and one reading *AIDS* pop up at the top of the screen. Man and woman, still surrounded by question marks, look at each other and then to the front. | El VIH y el SIDA están relacionados, pero no son la misma cosa. Déjame explicarte:  HIV and AIDS are related, but they are not the same thing. Let me explain. |
| 15. 1. Screen reads Para una i*nfección aguda de* VIH (in big purple letters) reciente  2. Underneath, middle of the screen appears a female cartoon character. She moves to the left of the screen and VIH flashes on her shirt. Six seconds later 3 a 6 semanas despues de la infeccion appear on the screen next to the female character. The female character will have an ice pack on her head and a thermometer in her mouth. VIH in large purple letters now appear over the female characters head and next to her appear the words *Síntomas* and underneath a list (in purple) that reads: *Fiebre, Pérdida de Peso, Sarpullido, Dolor de garganta, Gánglios linfáticos inflamados, Cansancio*  List disappears, *3 a 6 semanas despues de la infeccion* reappears.  *VIH* now moves to the middle of the screen, next to the character, above it the words *Infecction aguda de,* all words will slowly get bigger.  Then *esto no significa que tienes SIDA* appear at the bottom of the screen.  Then esto no significa que tienes SIDA disappears and the character loses the thermometer and the ice pack.  1. Screen reads *For a recent* *Acute HIV* (in big purple letters) *infection*  2. Underneath, middle of the screen appears a female cartoon character. She moves to the left of the screen and HIV flashes on her shirt. Six seconds later 3 to 6 weeks after the infection appear on the screen next to the female character. The female character will have an ice pack on her head and a thermometer in her mouth. HIV in large purple letters now appear over the female characters head and next to her appear the words *Symptoms* and underneath a list (in purple) that reads: *Fever, Weight Loss, Rash, Sore Throat, Swollen Lymph Nodes, Feel Tired*  List disappears, *3 to 6 weeks after the infection* reappears.  *HIV* now moves to the middle of the screen, next to the character, above it the words *An acute infection,* all words will slowly get bigger.  Then *this does not mean that you have HIV* appear at the bottom of the screen.  Then this does not mean that you have HIV disappears and the character loses the thermometer and the ice pack. | Immediatamente después de haberse infectado con el VIH, la mayoría de las personas no tienen ningun cambio en su cuerpo, o en cómo se sienten. Sin embargo, entre 3 y 6 semanas después, alguna de estas personas puede tener síntomas o señales tales como: fiebre (quick pause), pérdida de peso (quick pause), un sarpullido (quick pause), dolor de garganta (quick pause), ganglios linfáticos inflamados (quick pause), y cansancio (quick pause).  Si desarrollas estos síntomas o señales de 3 a 6 semanas después de haberte expuesto al VIH, quiere decir que tienes una infección aguda de VIH.  Una infección aguda de VIH es la reacción inicial de tu cuerpo al ser infectado con el VIH. Sin embargo, estos síntomas o señales no significa que tengas SIDA. Aunque te recuperarás de una infección aguda de VIH en un par de semanas, el VIH se quedara en tu cuerpo para siempre.  Immediately after being infected with HIV, most people do not feel any changes in their bodies or in how they feel. However, 3 to 6 weeks later some people can have symptoms or signs such as: fever (quick pause), weight loss (quick pause), a rash (quick pause), a sore throat (quick pause), swollen lymph nodes (quick pause) and feel tired (quick pause).  If you develop these symptoms or signs 3-6 weeks after being exposed to HIV that means you have an acute HIV infection.  An acute HIV infection is your body’s initial reaction to HIV. However, these symptoms or signs do not mean you have AIDS.  Even though you will recover from an acute HIV infection within a few weeks, HIV will stays in your body forever. |
| 16.  Show image of female cartoon with immune system being destroyed, show this image as it looked when it ended on frame 12 above. Several words “enfermedades” travel through the gaps in her immune system line. Word on the shirt of female cartoon reads VIH.  Place an hourglass with sand passing through on opposite side of screen, the passing of time should occur as the words are traveling through screen  Show image of female cartoon with immune system being destroyed, show this image as it looked when it ended in frame 12 above. Several words “*diseases*” travel to reach the female cartoon through the gaps in her immune system line. Word on the shirt of female cartoon still reads *HIV*.  Place an hourglass with sand passing through on opposite side of screen, the passing of time should occur as the words are traveling through screen | Como el VIH daña tu sistema inmunológico o defensas, con el TIEMPO te pueden dar enfermedades que un cuerpo sano podría normalmente combatir.  Because HIV damages your immune system or defenses, over TIME you can get diseases that your healthy body could normally fight off. |
| 17.  Female cartoon from above with words “*enfermedades*” entering through the gaps in her immune system line. As soon as the words reach her, the word *VIH* on her shirt changes to *SIDA*.  Side of the screen reads SIDA *en 5 a 10 años*  Female cartoon from above with words “*diseases*” entering through the gaps in her immune system line. As soon as the words reach her, the word *HIV* on her shirt changes to *AIDS*.  Side of the screen reads AIDS *in 5 to 10 years* | Cuando te dan estas enfermedades significa que tienes SIDA. Si tienes VIH y NO recibes el tratamiento que necesitas, te puede tomar entre 5 y 10 años desarrollar SIDA. (pause)  When you get these diseases, that means you have AIDS. If you have HIV and DO NOT get the treatment you need, it can take 5 to 10 years for you to develop AIDS. (pause) |
| 18.  1. Dissolve to cartoon of man and woman from before standing together as a couple.  2. The cartoon of a man wearing a green sweater that reads, *VIH* comes into the frame  When all three of them are on screen the top reads: Se pueden ver saludables  3. Couple moves out of the screen and the man with the VIH sweater remains alone on the screen, 4 people now enter the screen – a male and a female on each side of green sweater man. The four characters are smaller in size and each have a callout bubble that read ¿VIH?  Lastly top of the screen reads: Puede tener VIH y no lo sabe (2 lines)  1. Dissolve to cartoon of man and woman from before standing together as a couple.  2. The cartoon of a man wearing a green sweater that reads, *HIV* comes into the frame  When all three of them are on screen the top reads: May look healthy  3. Couple moves out of the screen and the man with the HIV sweater remains alone on the screen, 4 people now enter the screen – a male and a female on each side of green sweater man. The four characters are smaller in size and each have a callout bubble that read HIV?  Lastly top of the screen reads: Can have HIV and not know it (2 lines) | Las personas infectadas con el VIH o que tienen SIDA pueden verse completamente saludables. Es posible que no se vean enfermas. De hecho, estas personas se pueden ver igual que las personas que no tienen VIH o SIDA. Hay MUCHAS personas que están infectadas con el VIH y NO LO saben.  People who are infected with HIV or have AIDS can appear completely healthy. They might not look sick. In fact, they can look like people who do not have HIV or AIDS. There are MANY people who are infected with HIV and DO NOT know it. |
| 19.  The callout bubbles disappear and all four characters from above now have VIH across their shirts  The callout bubbles disappear and all four characters from above now have HIV across their shirts | Pero, SON portadoras del virus y pueden infectar a otras personas.(pause)  But, they ARE carrying the virus and can infect others.(pause) |
| 20.  Dissolve to two photos of real people, one showing a group of diverse young people together at a park, and the other one a photo of an older couple.  Top of the screen reads, *Cualquier persona se puede infectar con el VIH.*  Dissolve to two photos of real people, one showing a group of diverse young people together at a park, and the other one a photo of an older couple.  Top of the screen reads, *Anyone can get infected with HIV.* | Cualquier persona se puede infectar con el VIH.  Anyone can get infected with HIV. |
| 21.  Dissolve to two photos of male and female same sex couples, then changes to a photo of a hand injecting an arm and next to it a photo of a smiling older couple. Top of the screen reads, *Cualquier persona se puede infectar con el VIH*  Dissolve to two photos of male and female same sex couples, then changes to a photo of a hand injecting an arm and next to it a photo of a smiling older couple. Top of the screen reads, *Anyone can get infected with HIV* | NO IMPORTA cuan joven o viejo seas, si eres hombre o mujer, heterosexual o gay, o si usas drogas o no.  It doesn’t matter how old or how young you are, or if you are male or female, straight or gay, a drug user or a non-drug user. |
| 22.  Animation: two lines of people-shaped cut-outs moving into the screen one coming from the left, one coming from the right. After these rows move in, each character shows the color of a Latin American flags + Spain. The flags change a few times to show and represent all countries in Latin America.  Top of the screen reads *No importa quien seas.* On the next frame this phrase stays on top and an additional phrase appears at the bottom reading: *¡Lo que haces si importa!*  Animation: 1 globe with diverse group of cartoon characters dancing around it,  Top of the screen reads *It doesn’t matter who you are.* On the next frame this phrase stays on top and an additional phrase appears at the bottom reading: *What you do matters!* | NO IMPORTA quién seas o de dónde vengas. LO QUE IMPORTA es lo que haces y con quién lo haces. (pause)  It doesn’t matter who you are or where you are from. But IT DOES MATTER what you do and who you do it with. (pause) |
| 23.  Cartoon of young man and woman from before. Man has the word *VIH* on his sweater and it travels to the woman’s sweater, flickers and then disappears.  Cartoon of young man and woman from before. Man has the word *HIV* on his sweater and it travels to the woman’s sweater, flickers and then disappears. | El VIH se transmite, o pasa de una persona que tiene VIH a una persona que no lo tiene.  HIV is transmitted, or passed, from someone who has HIV to someone who does not have it. |
| 24.  New man/woman cartoon couple appears.  New man/woman cartoon couple appears. | El VIH se puede transmitir de hombre a mujer y de mujer a hombre  HIV can be passed between men and women. |
| 25.  Male cartoon couple appears.  Male cartoon couple appears. | De hombre a hombre  Between men. |
| 26.  Female cartoon couple appears.  Female cartoon couple appears. | Y de mujer a mujer. (pause)  And between women. (pause) |
| 27.  1. Cartoon couple looks up to words *NO PUEDES (in blue) infectarte con el VIH a través de:*  2. List of fluids that do not carry HIV appears. From top to bottom it reads, *Saliva, sudor, lágrimas, orina*.  List of words disappear, couple comes closer together and look at each other – male coughs and sneezes with tissue in hand and brings close to face – female has a callout bubble that says *pobrecito*  1. Cartoon couple looks up to words  *You CANNOT (in blue) get HIV from:*  2. List of fluids that do not carry HIV appears. From top to bottom it reads,  *Saliva, sweat, tears, urine*  List of words disappear, couple comes closer together and look at each other – – male coughs and sneezes with tissue in hand and brings close to face – female has a callout bubble that says *poor baby* | NO puedes infectarte con el VIH a través de la saliva (quick pause), el sudor (quick pause), las lágrimas (quick pause), la orina o sí alguien tose o estornuda a tu alrededor  You CANNOT get infected with HIV from saliva (quick pause), sweat (quick pause), tears (quick pause), urine or if someone coughs or sneezes around you. |
| 28.  Cartoon of a mosquito and another bug inside a crossed-out circle. Screen reads  *NO PUEDES (in blue) infectarte con el VIH a través de picaduras*  Cartoon of a mosquito and another bug inside a crossed-out circle. Screen reads  *You CANNOT (in blue) get HIV from insect bites* | TAMPOCO puedes infectarte con el VIH a través de las picaduras de mosquito o de cualquier otro insecto, aunque piquen a una persona con VIH Y DESPUES TE PIQUEN A TI.(pause)  You CANNOT get infected with HIV through bites from mosquitoes or any other insects even if they bite someone with HIV AND THEN BITE YOU .(pause) |
| 29.  Cartoon man and woman from before appear again and torsos get closer to each other and smile at each other, then hug and finally hold hands and kiss as hearts fly in the background  Top of the screen that reads: *El contacto diario es seguro*  Cartoon man and woman from before appear again and torsos get closer to each other and smile at each other, then hug and finally hold hands and kiss as hearts fly in the background  Top of the screen that reads: *Everyday contact is safe* | El VIH NO se transmite a través del contacto diario. Por lo tanto, abrazar, compartir un baño, darse la mano, compartir comida o bebidas y besar NO transmiten el VIH. (pause)  HIV is NOT transmitted through everyday contact. So hugging, sharing a bathroom, shaking hands, sharing food or drinks and kissing do NOT transmit HIV. (pause) |
| 30.  1. Cartoon couple looks up to words *PUEDES (in pink) infectarte con el VIH a través de:*  2. List of fluids that carry HIV appears. From top to bottom it reads, *semen, pre-eyaculación, fluidos vaginales, sangre, leche materna*.  1. Cartoon couple looks up to words  *You CAN (in pink) get HIV from:*  2. List of fluids that carry HIV appears. From top to bottom it reads, *semen, pre-ejaculate, vaginal fluids, blood, breast milk* | Te puedes infectar con el VIH a través de la sangre (quick pause), el semen (quick pause), la pre-eyaculación (quick pause), los fluídos vaginales – aun si la mujer no esta menstruando - (quick pause) o la leche materna.(pause)  You can get infected with HIV from blood (quick pause), semen (quick pause), pre-ejaculate (quick pause), vaginal fluids – even if a woman is not having her period - (quick pause), or breast milk.(pause) |
| 31.  1. Cartoon couple is on the screen. Man winks at woman  2. Callout from her saying ¡*NO (bold caps) sin un condón (bold)!*  3. Callout coming from him saying *Está bien*  1. Cartoon couple is on the screen. Man winks at woman  2. Callout from her saying *NOT (bold caps) without a condom! (bold)*  3. Callout coming from him saying *Ok* | Te PUEDES infectar con el VIH al tener sexo vaginal o anal SIN usar condón  You CAN get infected with HIV through vaginal or anal sex WITHOUT a condom. |
| 32.  Dynamic picture of hands sharing a syringe. Top of the screen reads, *PELIGRO*, *Compartir agujas o jeringuillas*  Dynamic picture of hands sharing a syringe. Top of the screen reads, *DANGER Sharing needles or syringes* | Y también te puedes infectar con el VIH al compartir jeringuillas o agujas cuando te inyectas cocaína, metanfetamina cristal, heroína, hormonas o esteroides. (pause)  You can also get infected with HIV when you share needles or syringes to inject drugs such as cocaine, heroin, crystal meth, hormones or steroids. (pause) |
| 33.  1. Fade into close-up picture of a jean pocket containing a red condom packet that is open and has a condom peaking out  2. Picture of a group of young people gathered at railroad tracks close to an area with a few used needles and condoms.  3. Top of the screen reads,  *PELIGRO*, *Compartir agujas o jeringuillas*  4. Fade into picture of hands holding a yellow condom.  Top of the screen reads, *PELIGRO, Tener sexo sin condón (vaginal o anal)*  1. Fade into close-up picture of a jean pocket containing a red condom packet that is open and has a condom peaking out  2. Picture of a group of young people gathered at railroad tracks close to an area with a few used needles and condoms.  3. Top of the screen reads, *DANGER Sharing needles or syringes*  4. Fade into picture of hands holding a yellow condom.  Top of the screen reads, *DANGER Sex without a condom (vaginal or anal)* | Mientras más a menudo tengas sexo vaginal o anal SIN condón o compartas agujas o jeringuillas más posibilidades tendrás de infectarte con el VIH. Pero recuerda, te puedes infectar con el VIH si tienes sexo SIN condón o compartes agujas tan solo UNA vez.  The more often you have vaginal or anal sex WITHOUT a condom or share needles or syringes the greater your chances of getting infected with HIV.  But remember, you can get infected with HIV if you have sex WITHOUT a condom or share needles even just ONE time. |
| 34.  Cartoon picture of a small baby with arrows pointing to it that read VIH durante el *embarazo y al nacer y VIH en la lactancia*  Cartoon picture of a small baby with arrows pointing to it that read *HIV during pregnancy and birth and HIV breastfeeding* | Una mujer infectada con el VIH, le puede transmitir el VIH a su bebé durante el embarazo, el parto o la lactancia. (pause)  A woman who is infected with HIV can pass HIV to her baby during pregnancy, birth or breastfeeding. (pause) |
| 35.  Screen reads, *Una infección de VIH se puede prevenir.*  Screen reads, *An HIV infection can be prevented* | Una infección de VIH se puede prevenir.  An HIV infection can be prevented |
| 36.  1. *Una infección de VIH se puede prevenir* goes to the top of the screen.  2. A picture of a notepad that reads, *Cenar con Juan Pablo,¡Comprar condones!* There is a condom and some earrings by the notepad.  3. Bottom of the screen reads, *Usar condones o no tener sexo.*  4. Bottom of the screen reads, *Menos parejas sexuales*  1. *An HIV infection can be prevented* goes to the top of the screen.  2. A picture of a notepad that reads, *Dinner with John, Buy condoms!* There is a condom and some earrings by the notepad.  3. Bottom of the screen reads,  *Use condoms or do not have sex*  4. Bottom of the screen reads, *Fewer sexual partners* | El VIH se puede prevenir usando condones durante el sexo (pause), no teniendo sexo (pause), o teniendo menos parejas sexuales. (pause)  HIV can be prevented by using condoms during sex (pause), not having sex (pause), or having fewer sexual partners. (pause) |
| 37.  Title: Situaciones Difíciles  Image of male protagonist here surrounded by a bottle of wine, glass of beer, medicine capsules (prescription drugs) and coins and bills that with the word “*DINERO*” in the center of them.  Character looks dizzy, with spiral lines revolving around his head to make him seem intoxicated. A callout bubble pops from his head reading ¡*Ay caramba! ¡Se me olvidó usar condones!*  Title: Difficult Situations  Image of male protagonist here surrounded by a bottle of wine (bottle reads WINE), a glass of beer, medicine capsules (prescription drugs) and coins and bills that with the word “*MONEY*” in the center of them.  Character looks dizzy, with spiral lines revolving around his head to make him seem intoxicated. A callout bubble pops from his head reading *Oh no! I forgot to use condoms!]* | Hay situaciones en las que se te puede ser difícil recordar usar condones. Por ejemplo, cuando…   - Tomas alcohol o usas drogas - Vendes sexo o pagas por tener sexo - Usas drogas o medicamentos que son estimulantes sexuales.   Por eso, recuerda ser MÁS cuidadoso en estas situaciones. (pause)  There are situations that make it hard for you to remember to use condoms. Such as:   - Drinking alcohol or using drugs - Selling or buying sex - Taking drugs or medications that are sexual stimulants.   So, remember to be extra careful during these times. (pause) |
| 38.  Cartoon man and woman are on the screen. Shared callout coming from both of them reads *Tengo que decirte algo*. A second shared callout appears reading *¡Tenemos que protegernos!*  Cartoon man and woman are on the screen. Shared callout coming from both of them reads *I have to tell you something*. A second shared callout appears reading *We have to protect ourselves!* | Al hablar con tus parejas sexuales sobre el VIH puedes saber si están infectadas o en riesgo de contraer VIH.  By talking to your sexual partners about HIV you can find out if they are infected or at risk for HIV. |
| 39.  Image of cartoon protagonists (male and female) one on each side of the screen. Next to each of them there are three opposite sex cartoons connected by a dotted line to the male and female respectively. A shared callout emerges from the heads of both characters that reads *¿Y si mi pareja anterior tenía VIH o SIDA?*  Image of cartoon protagonists (male and female) one on each side of the screen. Next to each of them there are three opposite sex cartoons connected by a dotted line to the male and female respectively. A shared callout emerges from the heads of both characters that reads *What if my last partner had HIV or AIDS?* | Pero ten en cuenta que puede que tus parejas sexuales no te digan si tienen VIH. (Pause)  O, puede que tus parejas sexuales no sepan si tienen VIH Y se vean completamente saludables. (Pause)  También es posible que tus parejas sexuales no sepan si las personas con las que tuvieron sexo en el pasado tenían VIH. (Pause)  However, keep in mind that your sexual partners might not tell you if they have HIV. (Pause)  Or, your sexual partners might not know if they have HIV and they might look completely healthy. (Pause)  Also, your sexual partners might not know if the people they had sex with in the past had HIV. (Pause) |
| 40.  . *Una infección de VIH se puede prevenir* is at the top of the screen  2. A picture of two syringes with a blue background appears. Place an “X” sign over this image  3. A picture of a new looking syringe with capping on top of needle and on the back of the syringe appears.  4. Bottom of the screen reads, No inyectes drogas  1.*HIV infection can be prevented*  is at the top of the screen  2. A picture of two syringes with a blue background appears. Place an “X” sign over this image  3. A picture of a new looking syringe with capping on top of needle and on the back of the syringe appears.  4. Bottom of the screen reads, *Do not inject drugs*  1. *Una infección de VIH se puede prevenir* is at the top of the screen  2. A picture with two hands sharing a syringe appears. A NO sign (red circle with a line) appears on top of the hands  3. Bottom of the screen reads, *Nunca compartas agujas o jeringuillas*  1. *HIV infection can be prevented* is at the top of the screen  2. A picture with two hands sharing a syringe appears. A NO sign (red circle with a line) appears on top of the hands  3. Bottom of the screen reads, *Never share needles or syringes.* | Tu también puedes evitar infectarte al no inyectarte drogas. Pero si te inyectas drogas usa agujas o jeringuillas limpias y nunca las compartas.  You can also prevent getting infected with HIV by not injecting drugs. But if you inject drugs use clean needles or syringes and never share them. |
| 41.  1.  *Una infección de VIH se puede prevenir* is at the top of the screen  2. A picture of a syringe submerged on a cup of water with a bottle of bleach in the background appears  1.*HIV infection can be prevented*  is at the top of the screen  2. A picture of a syringe submerged on a cup of water with a bottle of bleach in the background appears | Como último recurso, el cloro o blanqueador puede ayudarte a remover el VIH de agujas o jeringuillas antes de usarlas.(pause)  As a last resort, bleach might help remove HIV from used needles and syringes before they are used again.(pause) |
| 42.  1^st^ title: ¿*Entonces, por qué deberías hacerte la prueba del VIH?* Image of the cartoon couple protagonists of the video, looking at each other quizzically and surrounded by question marks. 2^nd^ title: *La unica manera de saber es haciéndote la prueba*  1^st^ title: *So, why should you be tested for HIV?* Image of the cartoon couple protagonists of the video, looking at each other quizzically and surrounded by question marks. 2nd title: *The only way to know is to be tested* | ¿Entonces, por qué deberías hacerte la prueba del VIH? (quick pause)  Porque LA ÚNICA manera de saber si tienes VIH es haciéndote la prueba.  So, why should you get tested for HIV? (quick pause)  Because THE ONLY way to find out if you have HIV is to be tested. |
| 43.  Live image of female medical professional, then transitions to live image of several medical professionals.  Live image of female medical professional, then transitions to live image of several medical professionals. | Los profesionales médicos recomiendan que te hagas la prueba del VIH en caso de que te hayas expuesto al VIH y no lo sepas.  Medical professionals recommend that you get tested for HIV just in case you were exposed to HIV and do not know it. |
| 44.  Image of male cartoon character then two female cartoon characters appear on screen, two dotted lines will connect male character to the female characters, the word ¿VIH? Flash on the female characters shirts. Then two male cartoon characters appear next to the female characters (one next to each) a dotted line connects each female to male character. The male characters shirts will also flash *¿VIH?* On their shirts.    Image here of male and female cartoon characters surrounded by question marks and with their shirts reading HIV? Flash on the female characters shirts. Then two male cartoon characters appear next to the female characters (one next to each) a dotted line connects each female to male character. The male characters shirts will also flash *HIV?* On their shirts. | Además, puede que no sepas si las personas con las que estas teniendo sexo o compartiendo agujas tienen VIH, o si están teniendo sexo o compartiendo agujas con alguien más.  Also, you may not know if the people you are having sex or sharing needles with have HIV, or if they are having sex or sharing needles with someone else. |
| 45.  1. Male cartoon character on screen, with question marks floating around and on his shirt. The question mark on his shirt then becomes a quickly flashing VIH and then changes to a steady VIH  2. He smiles and a callout with a stick figure exercising pops up from his head as well as one with a cut out of him talking to a doctor. Callout with image representing family/those who depend on you.  1. Male cartoon character on screen, with question marks floating around and on his shirt. The question mark on his shirt then becomes a quickly flashing HIV and then changes to a steady HIV  2. He smiles and a callout with a stick figure exercising pops up from his head as well as one with a cut out of him talking to a doctor. Callout with image representing family/those who depend on you. | Cuando el VIH se diagnostica y trata a tiempo puedes cuidarte y mantenerte saludable por más tiempo. Al mantenerte saludable ayudas a tu familia y a los que dependen de ti.  When HIV is diagnosed and treated early, you can work to stay healthy longer. Staying healthy can help your family and those who depend on you. |
| 46.  1. Cartoon man is in the center of the screen  2. Figures of doctors wearing green gowns and holding papers surround him  3. Top of the screen reads, *El tratamiento médico que necesitas* and then *No recibir tratamiento puede causar serios problemas de salud*  1. Cartoon man is in the center of the screen  2. Figures of doctors wearing green gowns and holding papers surround him  3. Top of the screen reads, *The medical treatments you need* and then  *Not getting treatment can cause serious health problems* | Al decirle a tus doctores que tienes VIH, puedes recibir el tratamiento médico que necesitas. Si el VIH NO se trata puede causar serios problemas de salud.  By telling your doctors you have HIV you can get the medical treatment you need.  If HIV is left UNTREATED, it can cause serious health problems. |
| 47.  1. Photo of a young pregnant woman on the right side of the screen.  Left side of the screen reads: *Protege a tu bebé*  2. Photo changes to show a picture of a pregnant belly.  3. Next to photo screen reads, *Durante el embarazo, hay medicamentos que pueden ayudar a reducir el riesgo de transmitir el VIH a tu bebé*  1. Photo of a young pregnant woman on the right side of the screen.  Left side of the screen reads *Protect your baby*  2. Photo changes to show a picture of a pregnant belly.  3. Next to photo screen reads,  *During pregnancy, medications are available to reduce the risk of passing HIV to your baby* | Si estás embarazada y estás infectada con el VIH, hay medicamentos que puedes tomar prevenir que tu bebé se infecte con el VIH .  If you are pregnant and are infected with HIV, there are medications you can take to prevent your baby from becoming infected with HIV. |
| 48.  1. Cartoon man with *VIH* word on sweater and cartoon woman are on the screen  2. Call out from cartoon man reads, *Tengamos sexo seguro*  3. Woman winks back and call out from her head reads, *Por supuesto, yo solo tengo sexo seguro*  1. Cartoon man with *HIV* word on sweater and cartoon woman are on the screen  2. Call out from cartoon man reads, *Lets have safe sex*  3. Woman winks back and call out from her head reads, *Of course, I only have safe sex* | También puedes tomar precauciones para prevenir infectar a otros, tales como usar condones y no compartir agujas o jeringuillas.  You can also take steps to prevent infecting others, such as using condoms and not sharing needles or syringes. |
| 49.  1. Carton man with *VIH* word on sweater is on the screen with cartoon woman.  2. Call out coming from cartoon man says to woman *Deberías hacerte la prueba de VIH*  3. Call out from her reads, *Por supuesto, me la hare*  1. Carton man with *VIH* word on sweater is on the screen with cartoon woman.  2. Call out coming from cartoon man says to woman *You should get tested for HIV*  3. Call out from her reads, *Of course, I will* | Puedes decirle a las personas con las que hayas tenido sexo o compartido agujas o jeringuillas que deben hacerse la prueba. (pause)  You can tell those you have had sex with or shared needles or syringes with that they should be tested. (pause) |
| 50.  Cartoon of woman meeting with a health professional.  Top of the screen a reads, *Las parejas deben hacerse la prueba*  Cartoon of woman meeting with a health professional.  Top of the screen a phrase that reads, *Partners should get tested* | En la mayoría de lugares hay servicios para notificar a las parejas que deben hacerse la prueba. (pause)  In most places, there are services to notify partners that they should be tested. (pause) |
| 51.  Screen reads, *Si tienes VIH*  Screen reads, *If you have HIV* | Enterarte de que estás infectado con el VIH puede causar un fuerte impacto en tu vida.  Learning that you are infected with HIV can have a strong impact on your life. |
| 52.  1. Word *ESTRÉS* in big bold letters appears on the screen.  2. Words *la espera* appears on top of the screen  3. Word  *ESTRÉS* stretches  1. Word *STRESS* in big bold letters appears on the screen.  2. Words *waiting* appears on top of the screen  3. Word *STRESS* stretches | Saber que estás infectado con el VIH puede ser estresante.  Knowing that you are infected with HIV can be stressful**.** |
| 53.  Words *sentirte solo* in big bold letters appear on the screen  Words *feeling alone* in big bold letters appear on the screen. | Las personas que se han enterado de que están infectadas con el VIH pueden sentirse aisladas o completamente solas.  People who have found out they are infected with HIV can feel isolated or all alone. |
| 54.  Word *problemas* is in the center of the screen in big bold letters, on top of this word appear *dinero o* *seguro médico* over a background of moving dollar signs  Word *problems* is in the center of the screen in big bold letters, on top of this word appear *money or* *insurance problems* over a background of moving dollar signs | También, puede que algunas personas con VIH tengan problemas de dinero, con el seguro médico, y más gastos médicos. (pause)  Also, some people with HIV might have problems with money, health insurance, or have more medical expenses. (pause) |
| 55.  Words *hay recursos disponibles para ayudarte* appear on the screen over a green background.  Words *resources are available* *to help you* appear on the screen over a green background. | Sin embargo, hay muchos recursos para ayudar a las personas con estos problemas.  However, there are many resources to help people with these problems. |
| 56.  Words *No hay una vacuna aprobada y no hay cura para el VIH* appear on the screen over a green background.  Words *There is no licensed vaccine and no cure for HIV* appear on the screen over a green background. | Aunque los científicos están trabajando en una vacuna para el VIH, no hay una vacuna lista y aprobada para prevenir el VIH. Y todavía NO HAY CURA.  Although scientists are working on a vaccine for HIV, there is no vaccine that is approved and ready to prevent HIV. And THERE STILL IS NO CURE. |
| 57.  Words *Hay tratamientos médicos disponibles para una vida larga y plena* appear on the screen over a green background.  Cartoon heads of the video characters look happy and surround the phrase. *Hay tratamientos médicos disponibles para una vida larga y plena*  Words *Medical treatments available for a longer, fuller life* appear on the screen over a green background.  Cartoon heads of the video characters looking happy and surrounding the phrase. *Medical treatments available for a longer, fuller life*  [Please make faces look more diverse in terms of ages and races; if possible representing all ethnic groups, races and ages i.e. American Indian, African American, Caucasian, Asian, old and young. | Pero, si te has infectado con el VIH, hay tratamientos médicos disponibles que te pueden ayudar a vivir una vida más larga y plena.(pause)  But, if you have been infected with HIV, there are medical treatments available to help you live a longer and fuller life. (pause) |
| 58  1. Top of the screen reads, *Pruebas de VIH*  2. Cartoon of many test tubes containing a pink fluid appear on screen.  3. *El tipo de prueba depende de tus necesidades médicas y de donde te hagas la prueba*  1. Top of the screen reads, *HIV tests*  2. Cartoon of many test tubes containing a pink fluid appear on screen.  3.  *Type of test depends on your medical needs and where you are tested.* | Hay muchas pruebas de VIH. El tipo de prueba que puedes recibir depende de tus necesidades médicas y de lo que esté disponible en el lugar donde te hagas la prueba.  There are many tests for HIV. The type of test you can receive depends on your medical needs and what is available where you are getting tested. |
| 59.  1. Top of the screen reads, *Prueba de VIH*  2. Cartoon of one test tube filled with a pink/red liquid next to the words *muestra de sangre*  3. On the right side, cartoon of one test tube filled with a purple liquid next to the words *muestra de fluidos orales*  Cartoon of test tube with purple oral fluid sample appears next to a cartoon of a mouth being swabbed next to the words *muestra oral*  1. Top of the screen reads, *HIV test*  2. Cartoon of one test tube filled with a pink/red liquid next to the words *blood sample*  3. On the right side, cartoon of one test tube filled with a purple liquid next to the words *oral fluid sample*  Cartoon of test tube with purple oral fluid sample appears next to a cartoon of a mouth being swabbed next to the words *oral fluid sample* | Puedes hacerte una prueba de VIH con una muestra de tu sangre o una muestra de tus fluídos orales.  You can be tested for HIV using a sample of your blood or a sample of your oral fluids. |
| 60.  1. Top of the screen reads, *Prueba de VIH*  2. Below plays a video that shows a lab tech getting blood from a man’s arm Scene shows a screen divided in two and showing the video currently part of this scene on one half (showing a regular blood draw) and on the other half insert a live image of a finger stick being performed.  1. Top of the screen reads, *HIV test*  2. Below plays a video that shows a lab tech getting blood from a man’s arm. Scene shows a screen divided in two and showing the video currently part of this scene on one half (showing a regular blood draw) and on the other half insert a live image of a finger stick being performed. | La muestra de sangre se saca de la misma manera como se saca para cualquier otro examen de sangre, (quick pause) o con un pinchazo en el dedo.  For a blood sample, blood is taken from you just like if you were getting any other blood test (quick pause) or with a finger stick. |
| 61.  Video of woman using a swab to collect an oral fluid sample from her mouth.  Video of woman using a swab to collect an oral fluid sample from her mouth. | La muestra de fluídos orales se saca usando un pequeño instrumento. (pause)  The oral fluid sample is collected using a small instrument. (pause) |
| 62.  Image of male cartoon protagonist next to a clock. Above his head bubble reads Esperando Resultados Time on clock is running, minute hand is rapidly moving and the clock changes color from yellow to green as the time passes. Cartoons shirt flashes VIH as time passes. Then top of screen reads Pruebas Rapidas de VIH*.*  Image of male cartoon protagonist next to a clock. Above his head bubble reads Waiting for Results Time on clock is running, minute hand is rapidly moving and the clock changes color, from yellow to green as the time passes. Cartoons shirt flashes HIV as time passes. Then top of screen reads Rapid HIV Tests*.* | Los resultados de algunas pruebas de VIH se pueden obtener el mismo día en el que te haces la prueba, usualmente en cuestión de minutos. Estas pruebas se conocen como pruebas rápidas de VIH.  For some HIV tests you can get your results the same day you got tested, usually within minutes. These tests are called rapid HIV tests. |
| 63.  Top of screen reads: Otras Pruebas de VIH  Two sets of images with this script.  - First, show male cartoon protagonist next to an arrow pointing to a tube filled with fluid similar to the one shown in scene 63 (this is his blood sample) and an arrow next to the tube pointing to the word *LABORATORIO*.  - Then, these images are replaced by the male under the cartoon of a calendar page which reads *MES* and has a day circled and reading *Prueba de VIH* and another day a week or two later circled and reading ¡*Resultado!*  *Cartoons shirts flashes VIH*  Top of screen reads: Other HIV Tests  Two sets of images with this script.  - First, show male cartoon protagonist next to an arrow pointing to a tube filled with fluid similar to the one shown in scene 63 (this is his blood sample) and an arrow next to the tube pointing to the word *LABORATORY*.  - Then, these images are replaced by the male under the cartoon of a calendar page which reads *MONTH* and has a day circled and reading *HIV test* and another day a week or two later circled and reading *Result!*  *Cartoons shirts flashes HIV* | Para OTRAS pruebas de VIH, tu sangre o fluídos orales se envían a un laboratorio para ser examinados. Para estas pruebas, es posible que tengas que esperar 1 o 2 semanas para obtener los resultados. También es posible que tengas que regresar al lugar donde te hiciste la prueba para saber tus resultados. (pause)  For OTHER HIV tests, your blood or oral fluid sample is sent to a laboratory for testing.  For these tests, you may need to wait 1 to 2 weeks to get the results. Also, you might have to return to the place where you got tested to get your results. (pause) |
| 64.  1. Cartoon male characters talking to a health professional  2. Top of the screen reads, *Es importante recibir los resultados*  3. Word *VIH* flashes on man’s shirt  1. Cartoon male characters talking to a health professional  2. Top of the screen reads, It is important *to get results*  3. Word *HIV* flashes on man’s shirt | Aunque esperar por tus resultados puede ser estresante, es MUY importante que recibas tus resultados para saber si tienes VIH. (pause)  Even though waiting for your results can be stressful, it is REALLY important that you get your results to know if you have HIV. (pause) |
| 65.  1. Blank calendar with word 1er Mes as title appear,each day is then crossed off with an X and the page flips to the next month. Title of second page is 2ndo Mes all days are crossed off with an X. Page then flips over to another calendar page with the title of 3cer Mes all days are crossed off with an X.  Top of the screen then reads Hasta tres meses para detectar el VIH.  . Blank calendar with word 1^st^ Month as title appear,each day is then crossed off with an X and the page flips to the next month. Title of second page is 2^nd^ Month all days are crossed off with an X. Page then flips over to another calendar page with the title of 3^rd^ Month all days are crossed off with an X.  Top of the screen then reads Up to three months to detect HIV | Hay otra cosa muy importante que debes saber sobre las pruebas de VIH.. Puede tomar hasta tres meses para que la prueba de VIH detecte la presencia de VIH en tu cuerpo después de que hayas infectado con el virus.  There is something else very important you should know about HIV testing. It can take an HIV test up to three months to detect the presence of HIV in your body after you become infected with the virus. |
| 66.  Cartoon of three pages of a calendar appear with first 2 pages flipped and 3cer Mes page showing.  Top of the screen reads Esto se conoce como el Período de Ventana  Cartoon of three pages of a calendar appear with first 2 pages flipped and 3rd Month page showing.  Top of the screen reads This is known as the Window Period | El tiempo que toma la prueba de VIH en detectar el VIH en tu cuerpo después de que te infectaste se conoce como el *período de ventana o tiempo de espera*.  The time is takes the HIV test to detect HIV in your body after you become infected is known as the window period. |
| 67.  Top of screen reads Período de Ventana  1. Cartoon torso of male and female protagonist are on the screen  2. Word *VIH* flashes on male cartoons shirt  3. Female cartoon character looks at the words on the male characters shirt.  4. The words VIH transitions on to the female cartoon characters shirt (both characters now have VIH on their shirt)  The words VIH flash as they appear on each characters shirts and then becomes steady.  Top of screen reads Window Period  1. Cartoon torso of male and female protagonist are on the screen  2. Word HIV flashes on male cartoons shirt  3. Female cartoon character looks at the words on the male characters shirt.  4. The words HIV transitions on to the female cartoon characters shirt (both characters now have VIH on their shirt)  The words HIV flash as they appear on each characters shirts and then becomes steady. | Durante el periodo de ventana, puede que sea muy temprano para que la prueba de VIH correctamente muestre que tienes VIH. Es decir, si recibes una prueba de VIH durante el periodo de ventana, tu prueba de VIH puede DECIR que no tienes VIH o que es negativo, aun cuando ESTAS infectado con VIH. .Pero si estás infectado con el VIH, puedes transmitírselo a otros durante este período. Por eso, es MUY importante que recuerdes usar condones y no compartas agujas o jeringuillas durante este tiempo.  During the window period, it may be too soon for the HIV test to correctly show that you have HIV. In other words, if you get an HIV test during the window period, your HIV test might say you are negative, even if you ARE infected with HIV. But if you are infected with HIV, you can transmit it to others during the window period. So, it is ESPECIALLY important to remember to use condoms and not share needles or syringes during this time. |
| 68.  Image showing first the male protagonist with callout bubbles that read 1) *Sexo sin condones, 2) Compartí agujas o jeringuillas* and 3) *Sexo con varias parejas.* Next we see him again, this time with just one callout bubble that reads *Quizás necesite otra prueba de VIH (purple) en el futuro*  Image to showing first the male protagonist with callout bubbles that read 1) *Sex without condoms, 2) Shared needles or syringes* and 3) *Sex with multiple partners.* Next we see him again, this time with just one callout bubble that reads  *May need another HIV(purple) test in the future* | Por esta razón, si en los últimos 3 meses hiciste cosas que pudieron ponerte en riesgo de infectarte con el VIH, es posible que necesites otra prueba en el futuro. (pause)  For this reason, if in the past 3 months you did things that could put you at risk for getting infected with HIV, you might need another HIV test in the future. (pause) |
| 69.  Cartoon of female character with a callout bubble that reads, ¿*Y si me infecté recientemente? Questions marks floating around in the background*  Cartoon of female character with a callout bubble that reads, *And if I got infected recently? Question marks floating around in the background* | En algunos casos, puedes obtener una prueba temprana o especial para ayudarte a saber más pronto si tienes VIH.  In some cases, you can get an early or special test to help you find out sooner if you have HIV. |
| 70  1.Top of the screen reads  *Pruebas especiales de VIH*  2. Underneath it reads *Para una infección de VIH* (in big purple letters) *reciente.*  3. Screen reads *Infección Aguda de* VIH (in big purple letters)  4. *Infección Aguda de VIH* moves to the top left corner and underneath appears a female cartoon character. Move cartoon character to middle of the screen, show her with an ice pack on her head and a thermometer in her mouth and show symptoms (words) floating around female character (*Fiebre, Pérdida de Peso, Sarpullido, Dolor de garganta, Gánglios linfáticos inflamados, Cansancio)*  1.Top of the screen reads  *Special or early HIV tests*  2. Underneath it reads *For a recent HIV* (in big purple letters) *infection.*  3. Screen reads *Acute HIV* (in big purple letters) *infection*  4. *Acute HIV infection* moves to the top left corner and underneath appears a female cartoon character. Move cartoon character to middle of the screen, show her with an ice pack on her head and a thermometer in her mouth and show symptoms (words) floating around female character (*Fever, Weight Loss, Rash, Sore Throat, Swollen Lymph Nodes, Feel Tired)* | Estas pruebas especiales o tempranas de VIH te pueden ayudar a saber si tienes una “infección aguda de VIH.”  Recuerda una infección aguda de VIH es la reacción inicial del cuerpo de 3 a 6 semanas después de haberse infectado con el VIH.  These special or early tests can help you find out if you have an “acute HIV infection.”  Remember an acute HIV infection is the body’s initial reaction 3 to 6 weeks after being infected with HIV. |
| 71.  1. Cartoon couple, word *VIH* travels from the male to the female character.  3. Top of the screen read *Puedes necesitar una prueba especial*  1. Cartoon couple, word *HIV* travels from the male to the female character.  3. Top of the screen read *You might need a special test* | Si piensas que te infectaste con el VIH recientemente y tienes síntomas o señales de una infección aguda de VIH, es posible que necesites una prueba especial.  If you think you got infected with HIV recently, and have symptoms or signs of an acute HIV infection, you might need a special or early test for this. |
| 72.  Male protagonist speaking to healthcare provider.  Male protagonist speaking to healthcare provider. | Puedes preguntarle a tu proveedor medico si una prueba temprana o especial está disponible, y si es adecuada para ti. (pause)  You can ask your healthcare provider if a special or early HIV test is available and right for you. (pause) |
| 73.  1. Top of the screen reads, *Posibles Resultados*  2. The screen is divided vertically in four parts showing a -, +, +, ?.  Image shows only two possibilities + (in green) and – (in blue). One by one the words Negativo and Positivo appear underneath each sign.  1. Top of the screen reads, *Possible Test Results*  2. The screen is divided vertically in four parts showing a -, +, +, ?.  Image shows only two possibilities + (in green) and – (in blue). One by one the words negative and positive appear underneath each sign. | Hay 2 resultados para las pruebas de VIH: negativo y positivo.  There are 2 results for HIV tests: negative and positive. |
| 74.  1. Top of the screen reads, *Posibles Resultados*  2. Screen is blue with floating negative signs  3. Big blue bold negative sign in the middle in parenthesis with the word *Negativo* underneath and  4. Bottom of the screen shows the following phrases in order  - *No se detectó VIH*  - *Puede que sea demasiado pronto para detectar el VIH*  *-* *Hazte la prueba de nuevo en 3 meses para estar seguro*  *- Prueba especial para una infección aguda de VIH*  1. Top of the screen reads, *Possible Test Results*  2. Screen is blue with floating negative signs  3. Big blue bold negative sign in the middle in parenthesis with the word *Negative* underneath and  4. Bottom of the screen shows the following phrases in order  - *No HIV detected*  - It may be too *soon to detect HIV*  *-* *Retest in 3 months to be sure*  *- Special or early test for an acute HIV infection* | Un resultado negativo significa que no se encontró VIH en tu cuerpo. (pause)  Pero recuerda, debido al período de ventana puede que sea DEMASIADO PRONTO para que la prueba muestre si tienes VIH.(pause)  Por lo tanto, puede que necesites otra prueba de VIH en el futuro. (pause)  O, para enterarte si te has infectado recientemente, puede que necesites una prueba especial para una infección aguda de VIH.  A negative test result means that HIV was not found in your body. (pause)  But remember, because of the window period, it may be TOO SOON for the test to show if you have HIV. (pause)  So, you may need another HIV test in the future. (pause)  Or, to find out if you have been infected recently, you might need a special or early test for an acute HIV infection. |
| 75.  1. Top of the screen reads, *Posibles Resultados*  Two plus sings appear (+) side by side. On is yellow and one is purple. Underneath each sign appear the words Definitivo (under purple) and Positivo Preliminar (under yellow)  Top of the screen reads *Resultado Positivo*  Bottom of the screen shows the following phrases in order  1. Top of the screen reads, *Possible Test Results*  Two plus sings (+) side by side. On is yellow and one is purple. Underneath each sign appear the words Final (under purple) and Preliminary Positive (under yellow)  Top of the screen reads *Positive Result*  Bottom of the screen shows the following phrases in order. | Un resultado positivo de una prueba de VIH puede ser PRELIMINAR o DEFINITIVO, dependiendo de si te hiciste una prueba rápida de VIH o no.  A positive HIV test result can be PRELIMINARY or FINAL, depending upon whether or not you had a rapid HIV test. |
| 76.  Top of screen reads: Resultado de una prueba rápida de VIH  Protagonist male character with *¿VIH?* on his shirt, on side with screen with a clock next to him, minute hand starts at 12 and moves around the whole clock. Clock changes from green to yellow as it moves by and stops changing color when it gets to the 6 on clock.  Clipboard appears and slides over the clock, the clipboard reads RESULTADO DE PRUEBA DE VIH  the middle of the clipboard has a positive sign in between a parenthesis (+) under the sign reads POSITIVO PRELIMINAR  Top of screen reads: Rapid HIV test results  Protagonist male character with HIV on his shirt, on side with screen with a clock next to him, minute hand starts at 12 and moves around the whole clock. Clock changes from green to yellow as it moves by and stops changing color when it gets to the 6 on clock.  Clipboard appears and slides over the clock, the clipboard reads RAPID HIV TEST RESULTS  the middle of the clipboard has a positive sign in between a parenthesis (+)under the sign reads PRELIMINARY POSITIVE | Si te hiciste una prueba rápida de VIH y el resultado salió positivo, este es UNICAMENTE un resultado positivo preliminar. Un resultado positivo preliminar significa que PUEDE que estés infectado con el VIH y puedes infectar a los demás. Sin embargo, este no es un resultado final.  If you received a rapid HIV test and the result was positive, this is ONLY a preliminary positive test result. A preliminary positive test result means that you COULD be infected with HIV and could infect others. However, this is not the final answer. |
| 77.  Change image completely to show only clipboard from clip #76 place on middle of screen, larger. Then move to the upper left corner of the screen*.* Insert an arrow next to this paper, pointing to another clipboard that reads PRUEBA ADICIONAL DE VIH above and below these words appear + - + - on the other side another arrow, insert another clipboard on the other side of the arrow reading ¡*RESULTADO FINAL!*  *-*  Change image completely to show only clipboard from clip #76 place on middle of screen, larger. Then move to the upper left corner of the screen*.* Insert an arrow next to this paper, pointing to another clipboard that reads ADDITIONAL HIV TESTS above and below these words appear + - + - on the other side another arrow, insert another clipboard on the other side of the arrow reading FINAL RESULT*!* | Si el resultado de la prueba rápida de VIH es positivo preliminar, DEBE ser confirmado con una prueba adicional de VIH. La prueba adicional de VIH te dirá si estás infectado con VIH o no. Puede tomar una semana o más obtener los resultados finales. Es MUY importante que recibas tus resultados finales para que puedas saber si tienes VIH. (pause)  If the rapid HIV test result is preliminary positive, it MUST be confirmed with an additional HIV test. The additional test will tell you whether or not you are infected with HIV. It can take a week or more to get the final test results. It is very important for you to get your final results so you can find out if you have HIV. (pause) |
| 79.  [Please insert here previous image 78 showing a positive result with the word *POSITIVO* under a purple sign.]   - Top of the screen reads *Posibles Resultados* - Bottom of the screen reads   - *Tienes VIH*   - *Asegúrate de recibir tratamieto*   [Please insert here previous image 78 showing a positive result with the word *POSITIVE* under a purple sign.]   - Top of the screen reads *Possible Results* - Bottom of the screen reads   - *You have HIV*   - *Make sure to get treatment* | Para todos tipos de pruebas, un resultado positivo definitivo quiere decir que tienes VIH. Pero recuerda, esto significa que puedes trabajar con tus proveedores de salud para recibir el tratamiento que necesitas y para prevenir infectar a otros. (pause)  For all types of tests, a final positive test result means that you have HIV. But remember, this means that you can work with your health care provider to get the treatment you need and to prevent infecting others. (pause) |
| 80.  1. Top of the screen reads, *Se te explicarán tus resultados*  2. Cartoon woman talking to a health care professional. In the background floating question marks.  1. Top of the screen reads, *Your results will be explained to you*  2. Cartoon woman talking to a health care professional. In the background floating question marks. | Los resultados de tu prueba de VIH se te explicarán. Siéntete libre de hacer preguntas si no entiendes tus resultados. (pause)  Your HIV test results will be explained to you. Feel free to ask questions if you do not understand your test results. (pause) |
| 81.  Cartoon of clipboard where the following info appears and then a check appears next to after right after they are read by the narrator.  - *EL VIH es el virus que causa el SIDA*  *- El VIH se transmite a través del sexo sin protección o al compartir agujas o jeringuillas*  Cartoon of clipboard where the following info appears and then a check appears next to after right after they are read by the narrator.  - *HIV is the virus that causes AIDS*  *- HIV can be passed through sex without a condom or by sharing needles or syringes* | Aquí hay algunos puntos que recordar:   - El VIH es el virus que causa el SIDA. - El VIH se transmite de persona a persona a través del sexo sin condón o al compartir agujas o jeringuillas.   Here are a few points to remember:   - HIV is the virus that causes AIDS. - HIV can be transmitted from person to person through sex without a condom or by sharing needles or syringes. |
| 82.  New page on the clipboard that reads,:  *El VIH se previene al*:  *- Usar condones*  *- No tener sexo*  *- Usar agujas o jeringuillas limpias y al no compartirlas*  *- No inyectar drogas*  New page on the clipboard that reads,:  *HIV can be prevented by:*  *- Using condoms*  *- Not having sex*  *- Using clean needles and syringes or not sharing them*  *- Not injecting drugs* | El VIH se puede prevenir usando condones durante el sexo, no teniendo sexo, usando agujas o jeringuillas limpias o no compartiéndolas y no inyectando drogas.  HIV can be prevented by using condoms during sex, not having sex, using clean needles and syringes or not sharing them and not injecting drugs, |
| 83.  New page on clipboard that reads,:  *Si no recibiste tus resultados el mismo día en el que te hiciste la prueba:*   - *Asegúrate de recibir tus resultados*   *If you did not get your results the same day you got tested:*   - *Make sure to get your results* | Si no recibiste tus resultados el mismo día en el que te hiciste la prueba de VIH, asegúrate de recibirlos cuando estén disponibles.  If you did not get your test results the same day you got tested, make sure to get them when they are available. |
| 84.  Another image of the clipboard reading:  *Si tu prueba rapida de VIH es positiva preliminar:*   - *Necesitarás hacerte otra prueba para confirmar que tienes VIH*   *If your rapid HIV test is preliminary positive:*  - *You will need another test to confirm that you have HIV* | Si tu prueba rapida de VIH sale positiva preliminar, necesitarás una prueba adicional para confirmar los resultados.  If your rapid HIV test is preliminary positive, you will need an additional test to confirm the results. |
| 85.  1. Cartoon man and woman appear with a sign underneath that reads, *Para mayor información contactar* and below appear *Consejero de VIH* *Proveedor de salud* and then *Agencia de salud nacional o local*  1. Cartoon man and woman appear with a sign underneath that reads, *For more information contact* and below *appear HIV test counselor* *Health care provider* and then *Local or National Health Agency* | Para mayor información sobre las pruebas del VIH, pregúntale a un consejero que haga pruebas de VIH o contacta a tu proveedor de salud o a una agencia local o nacional de salud.  For more information about HIV testing ask your HIV test counselor or contact your health care provider or local or national health agency |
| 86.  Similar screen to the opening screen with background containing plus and minus signs. Screen reads, *Actúa con inteligencia. ¡Hazte la prueba del VIH!*    Similar screen to the opening screen with background containing plus and minus signs. Screen reads, Be Smart. Get Tested! | Music |
